# Publisher Correction: Immunoprophylactic and immunotherapeutic control of hormone receptor-positive breast cancer

**DOI:** 10.1038/s41467-020-18719-8

**Published:** 2020-09-17

**Authors:** Aitziber Buqué, Norma Bloy, Maria Perez-Lanzón, Kristina Iribarren, Juliette Humeau, Jonathan G. Pol, Sarah Levesque, Laura Mondragon, Takahiro Yamazaki, Ai Sato, Fernando Aranda, Sylvère Durand, Alexandre Boissonnas, Jitka Fucikova, Laura Senovilla, David Enot, Michal Hensler, Margerie Kremer, Gautier Stoll, Yang Hu, Chiara Massa, Silvia C. Formenti, Barbara Seliger, Olivier Elemento, Radek Spisek, Fabrice André, Laurence Zitvogel, Suzette Delaloge, Guido Kroemer, Lorenzo Galluzzi

**Affiliations:** 1 Department of Radiation Oncology, Weill Cornell Medical College, New York, NY USA; 2 Equipe labellisée par la Ligue contre le cancer, Centre de Recherche des Cordeliers, INSERM U1138, Université de Paris, Sorbonne Université, Paris, France; 3 Faculté de Médecine, Université de Paris Sud, Paris-Saclay, Le Kremlin-Bicêtre, Paris, France; 4 Metabolomics and Cell Biology Platforms, Gustave Roussy Comprehensive Cancer Institute, Villejuif, France; 5 Sorbonne Université, Inserm, CNRS, Centre d’Immunologie et des Maladies Infectieuses CIMI, Paris, France; 6 Sotio, Prague, Czech Republic; 7 Department of Immunology, Charles University, 2nd Faculty of Medicine and University Hospital Motol, Prague, Czech Republic; 8 Caryl and Israel Englander Institute for Precision Medicine, New York, NY USA; 9 Institute of Medical Immunology, Martin Luther University Halle-Wittenberg, Halle, Germany; 10 Sandra and Edward Meyer Cancer Center, New York, NY USA; 11 Gustave Roussy Cancer Center, Villejuif, France; 12 INSERM, U1015 Villejuif, France; 13 Center of Clinical Investigations in Biotherapies of Cancer (CICBT) 1428, Villejuif, France; 14 Department of Cancer Medicine, Gustave Roussy Cancer Center, Villejuif, France; 15 Pôle de Biologie, Hôpital Européen Georges Pompidou, AP-HP, Paris, France; 16 Suzhou Institute for Systems Medicine, Chinese Academy of Medical Sciences, Suzhou, China; 17 Department of Women’s and Children’s Health, Karolinska University Hospital, Stockholm, Sweden; 18 Department of Dermatology, Yale School of Medicine, New Haven, CT USA; 19 Université de Paris, Paris, France

**Keywords:** Breast cancer, Cancer prevention, Cancer therapy, Tumour immunology

Correction to: *Nature Communications* 10.1038/s41467-020-17644-0, published online 30 July 2020.

The original version of this Article contained an error in Fig. 1. In Fig. 1e, the label “*ER-WT* (*n* = 12)” was inadvertently duplicated. The red curve should have been labelled “*ER-AF2*^*0*^ (*n* = 12)”. This error has now been corrected in both the PDF and HTML versions of the Article.

