# Supplementary material for: Immunoprophylactic and immunotherapeutic control of hormone receptor-positive breast cancer
Source: Nat Commun. 2020 Jul 30;11:3819. doi: 10.1038/s41467-020-17644-0 (PMC7393498; doi:10.1038/s41467-020-17644-0)
Supplement: Supplementary file 1 — Supplementary Information [file 41467_2020_17644_MOESM1_ESM.pdf]

Immunoprophylactic and immunotherapeutic control of hormone-receptor positive breast cancer

Buqué et al.

Supplementary Figures

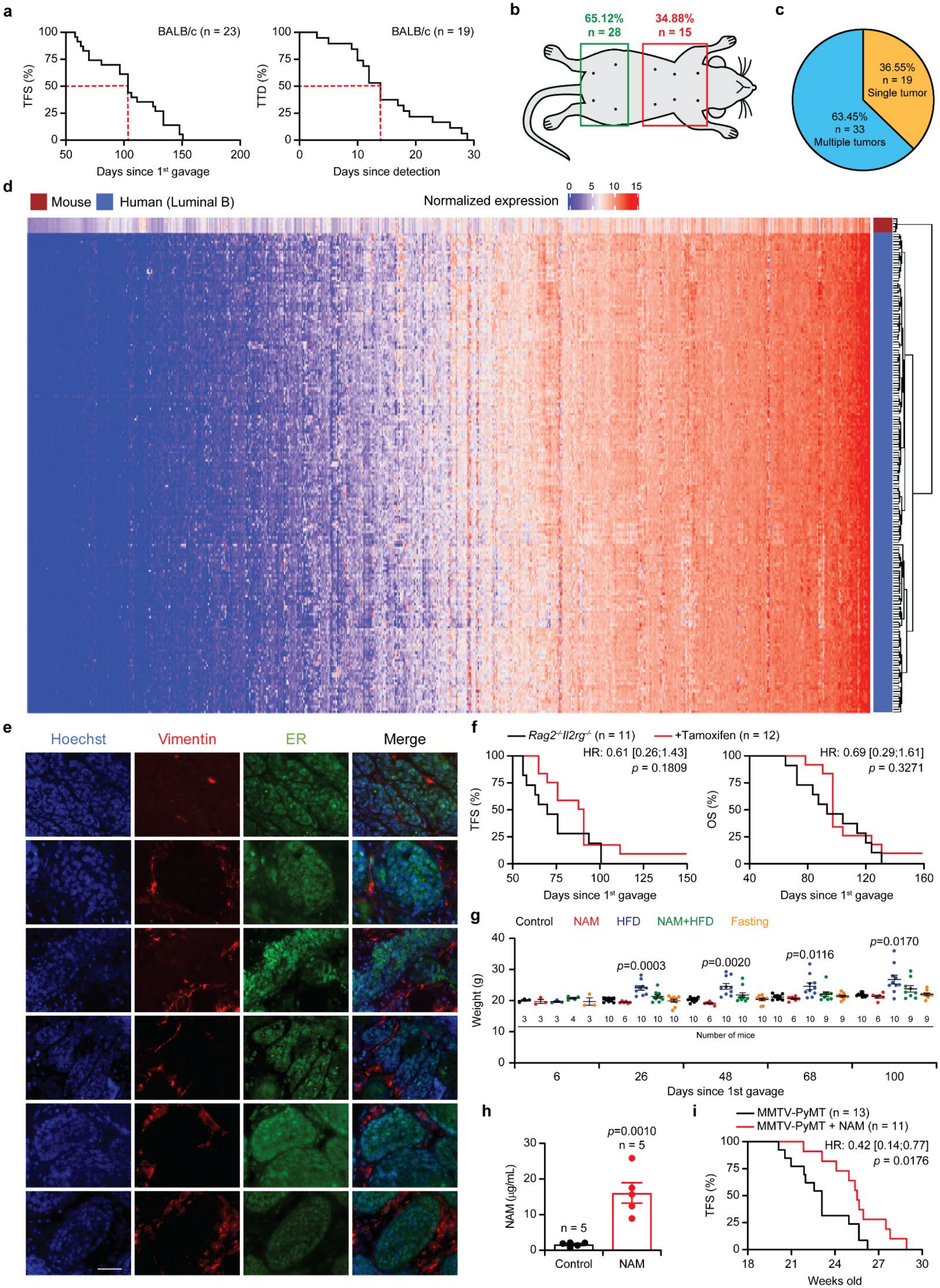
a
BALB/c (n = 23)
TFS (%)
Days since 1st gavage
BALB/c (n = 19)
TTD (%)
Days since detection
b
65.12%
n = 28
34.88%
n = 15
c
36.55%
n = 19
Single tumor
63.45%
n = 33
Multiple tumors
d
Mouse
Human (Luminal B)
Normalized expression
e
Hoechst
Vimentin
ER
Merge
f
Rag2-/-Il2rg-/- (n = 11)
+Tamoxifen (n = 12)
HR: 0.61 [0.26;1.43]
p = 0.1809
HR: 0.69 [0.29;1.61]
p = 0.3271
TFS (%)
OS (%)
Days since 1st gavage
g
Control
NAM
HFD
NAM+HFD
Fasting
p=0.0003
p=0.0020
p=0.0116
p=0.0170
Weight (g)
Number of mice
Days since 1st gavage
h
p=0.0010
n = 5
n = 5
NAM (μg/mL)
Control
NAM
i
MMTV-PyMT (n = 13)
MMTV-PyMT + NAM (n = 11)
HR: 0.42 [0.14;0.77]
p = 0.0176
TFS (%)
Weeks old

Supplementary Figure 1. **M/D-driven tumors as a model of HR$^+$ BC sensitive to nutritional interventions. a.** Tumor-free survival (TFS) and time-to-death (TTD) of WT BALB/c mice subjected to M/D-driven oncogenesis. Number of mice is reported. **b.** Anatomical localization of M/D-driven tumors evolving in WT C57BL/6 mice. **c.** Percentage of multifocal *vs* focal disease upon M/D-driven carcinogenesis in WT C57BL/6 mice. **d.** Hierarchical clustering of orthologous human and mouse genes from M/D-driven tumors evolving in WT C57BL/6 mice (n = 6) and luminal B breast cancer (BC) patients included in the TCGA database (n = 187). **e.** Molecular phenotype of M/D-driven tumors evolving in WT C57BL/6 mice, as assessed by immunofluorescence for estrogen receptor 1 (ESR1, best known as ER) and vimentin expression. Hoechst 33258 was employed for nuclear counterstaining. Scale bar = 40 µm. **f.** TFS and overall survival (OS) of *Rag2$^{-/-}$Il2rg$^{-/-}$* mice subjected to M/D-driven oncogenesis in control conditions or along with tamoxifen administration with the drinking water. Number of mice, hazard ratio (HR) and *p* values (two-sided log-rank) are reported. **g.** Body weight of C57BL/6 mice subjected to M/D-driven carcinogenesis in control conditions or in the context of the indicated nutritional interventions. Results are means ± SEM and individual data points. Number of mice and *p* values are reported (unpaired, two-sided Student's t, as compared to age-matched mice maintained in control conditions). HFD, high-fat diet. **h.** Circulating levels of NAM in C57BL/6 mice maintained in control housing conditions or receiving NAM supplementation with the drinking water for 14 days. Results are means ± SEM and individual data points. Number of mice and *p* value are reported (unpaired, two-sided Student's t). **i.** TFS of MMTV-PyMT mice maintained in control conditions or subjected to NAM supplementation with the drinking water. Number of mice, HR and *p* values (two-sided log-rank) are reported.

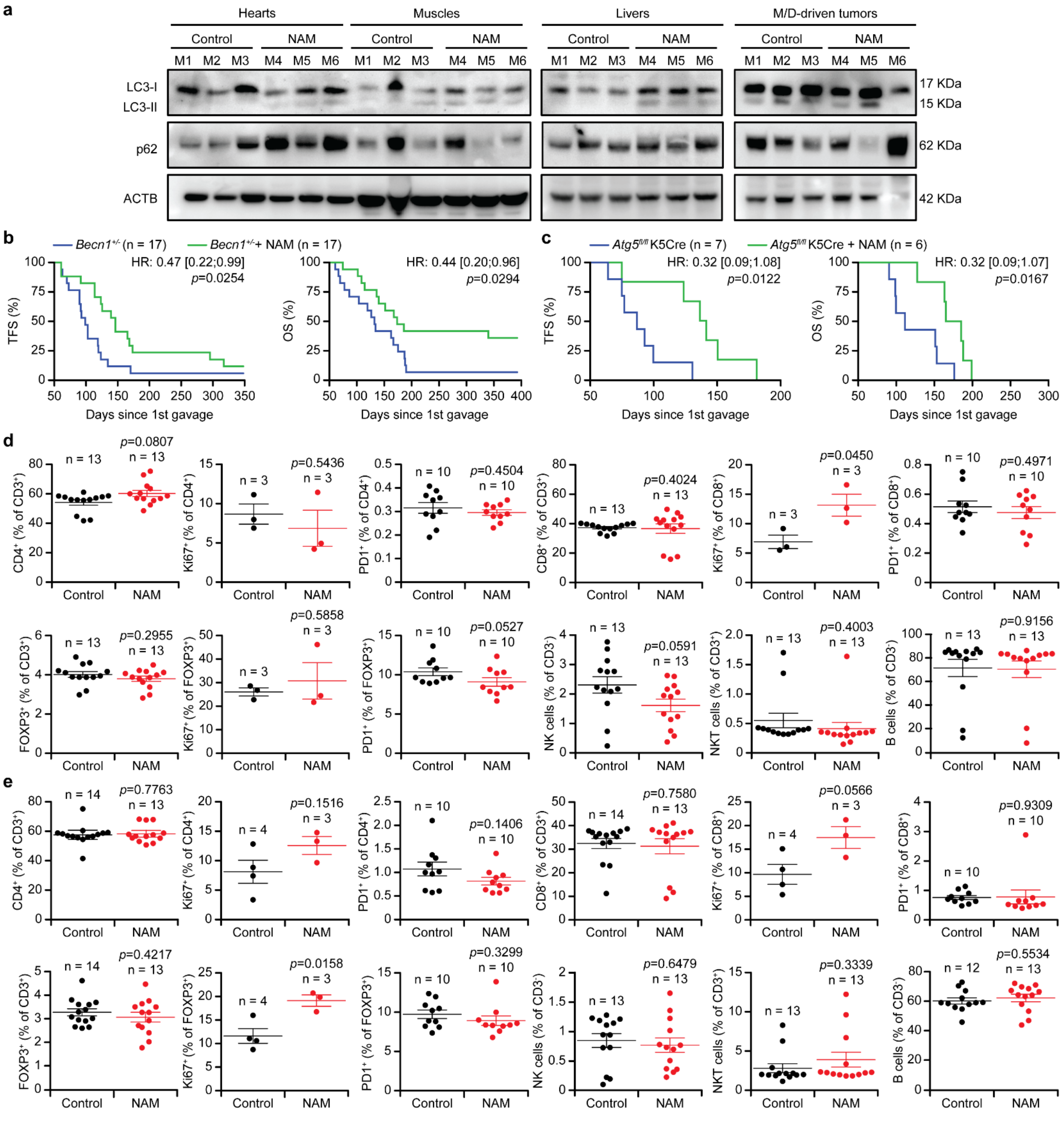

***Supplementary Figure 2.* Mechanisms of NAM-mediated oncoprevention. a.** LC3 lipidation and p62 levels in different organs from C57BL/6 mice subjected to M/D-driven carcinogenesis in control conditions or in the context of 0.5% w/w NAM supplementation with the drinking water. ACTB levels were assessed as loading control. Uncropped immunoblots are provided as a Source Data file. **b, c.** Tumor-free survival (TFS) and overall survival (OS) of *Becn1*$^{+/-}$ mice (**b**) and *Atg5*$^{fl/fl}$*KRT5-Cre* mice (**c**) subjected to M/D-driven oncogenesis in control conditions or along with 0.5% w/w NAM supplementation with the drinking water. Number of mice, hazard ratio (HR) and *p* values (two-sided log-rank) are reported. **d, e.** Relative abundance and proliferative potential (based on Ki67 positivity) of $CD8^+$ T cells, $CD4^+$ T cells, $CD4^+CD25^+FOXP3^+$ T cells, $CD3^-CD56^+$ NK cells, $CD3^+CD56^+$ NKT cells, and $CD19^+B220^+$ B cells in the spleen (**d**) and lymph nodes (**e**) of C57BL/6 mice maintained in control conditions or receiving 0.5% w/w NAM supplementation with the drinking water for 14 days. Results are means ± SEM and individual data points. Number of mice and *p* values (unpaired, two-sided Student's t) are reported.

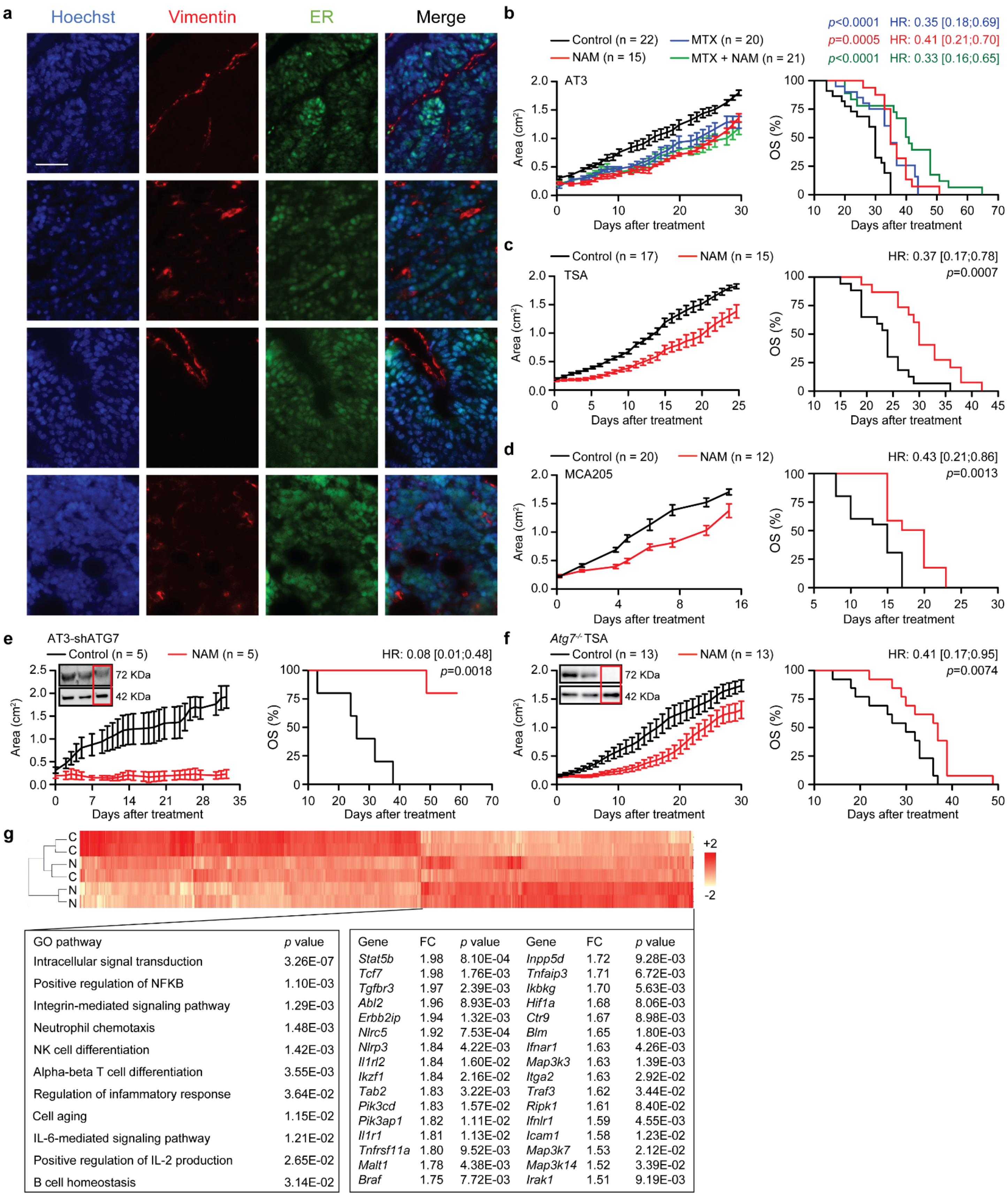

GO pathway	p value
Intracellular signal transduction	3.26E-07
Positive regulation of NFKB	1.10E-03
Integrin-mediated signaling pathway	1.29E-03
Neutrophil chemotaxis	1.48E-03
NK cell differentiation	1.42E-03
Alpha-beta T cell differentiation	3.55E-03
Regulation of infammatory response	3.64E-02
Cell aging	1.15E-02
IL-6-mediated signaling pathway	1.21E-02
Positive regulation of IL-2 production	2.65E-02
B cell homeostasis	3.14E-02

Gene	FC	p value	Gene	FC	p value
Stat5b	1.98	8.10E-04	*Inpp5d*	1.72	9.28E-03
Tcf7	1.98	1.76E-03	*Tnfaip3*	1.71	6.72E-03
Tgfbr3	1.97	2.39E-03	*Ikbkg*	1.70	5.63E-03
Abl2	1.96	8.93E-03	*Hif1a*	1.68	8.06E-03
Erbb2ip	1.94	1.32E-03	*Ctr9*	1.67	8.98E-03
Nlrc5	1.92	7.53E-04	*Blm*	1.65	1.80E-03
Nlrp3	1.84	4.22E-03	*Ifnar1*	1.63	4.26E-03
Il1rl2	1.84	1.60E-02	*Map3k3*	1.63	1.39E-03
Ikzf1	1.84	2.16E-02	*Itga2*	1.63	2.92E-02
Tab2	1.83	3.22E-03	*Traf3*	1.62	3.44E-02
Pik3cd	1.83	1.57E-02	*Ripk1*	1.61	8.40E-02
Pik3ap1	1.82	1.11E-02	*Ifnlr1*	1.59	4.55E-03
Il1r1	1.81	1.13E-02	*Icam1*	1.58	1.23E-02
Tnfrsf11a	1.80	9.52E-03	*Map3k7*	1.53	2.12E-02
Malt1	1.78	4.38E-03	*Map3k14*	1.52	3.39E-02
Braf	1.75	7.72E-03	*Irak1*	1.51	9.19E-03

***Supplementary Figure 3.* Mechanistic insights into the therapeutic activity of NAM. a.** Molecular phenotype of M/D-driven tumors established in WT C57BL/6 mice and progressing despite NAM supplementation with the drinking water, as assessed at ethical endpoint by immunofluorescence for estrogen receptor 1 (ESR1, best known as ER) and vimentin expression. Scale bar = 40 µm. **b-d.** Tumor growth and overall survival (OS) in C57BL/6 or BALB/c mice bearing established AT3 (**b**), TSA (**c**) or MCA205 (**d**) tumors that were left untreated or received NAM supplementation with the drinking water, intraperitoneal MTX treatment or both (as indicated). Tumor

growth results are means ± SEM. Number of mice, hazard ratio (HR) and *p* values (two-sided log-rank for OS) are reported. **e, f.** Tumor growth and OS in C57BL/6 or BALB/c mice bearing established ATG7-depleted AT3 tumors (**e**) or *Atg7*$^{-/-}$ TSA tumors (**f**) that were maintained in control conditions or subjected to NAM supplementation with the drinking water. Tumor growth results are means ± SEM. Number of mice, HR and *p* values (two-sided log-rank for OS) are reported. Insets depict ATG7 levels (top blots, 72 KDa) in ATG7-competent (wild type or control-transfected, left and central lanes) *vs* ATG7-deficient (right lanes) cells. Actin levels were monitored as loading control (bottom blots, 42 KDa). Uncropped immunoblots are provided as a Source Data file. **g.** Unsupervised hierarchical clustering of genes differentially expressed in untreated (n = 3) *vs* NAM-treated (n = 3) AT3 tumors. Top upregulated genes, fold change (FC) and adjusted *p* values are reported. Gene Ontology (GO) analysis and adjusted, two-sided *p* values for enrichment are indicated.

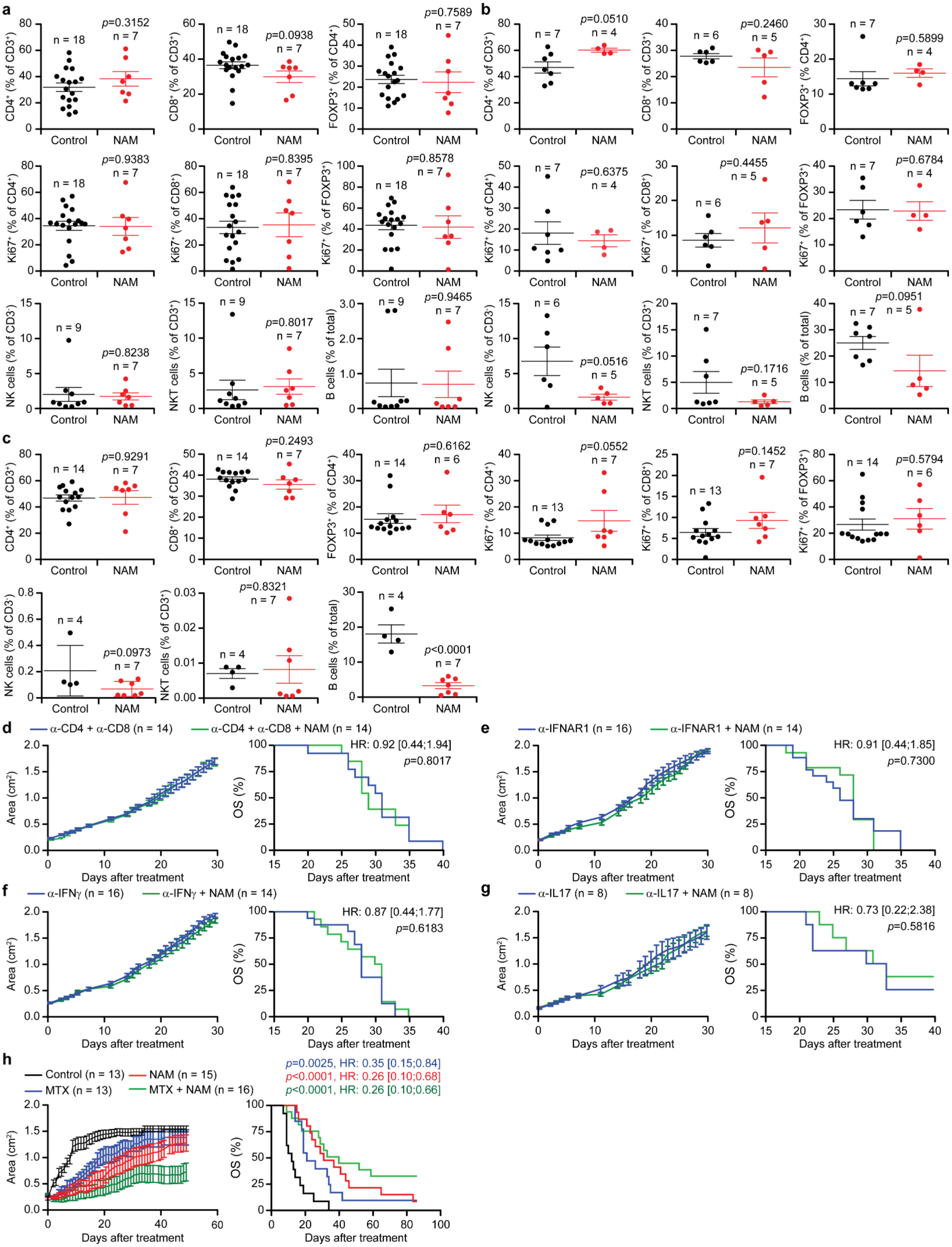
a
CD4⁺ (% of CD3⁺)
n = 18
p=0.3152
n = 7
Control
NAM
CD8⁺ (% of CD3⁺)
n = 18
p=0.0938
n = 7
FOXP3⁺ (% of CD4⁺)
n = 18
p=0.7589
n = 7
b
CD4⁺ (% of CD3⁺)
n = 7
p=0.0510
n = 4
CD8⁺ (% of CD3⁺)
n = 6
p=0.2460
n = 5
FOXP3⁺ (% CD4⁺)
n = 7
p=0.5899
n = 4
Ki67⁺ (% of CD4⁺)
n = 18
p=0.9383
n = 7
Ki67⁺ (% of CD8⁺)
n = 18
p=0.8395
n = 7
Ki67⁺ (% of FOXP3⁺)
n = 18
p=0.8578
n = 7
Ki67⁺ (% of CD4⁺)
n = 7
p=0.6375
n = 4
Ki67⁺ (% of CD8⁺)
n = 6
p=0.4455
n = 5
Ki67⁺ (% of FOXP3⁺)
n = 7
p=0.6784
n = 4
NK cells (% of CD3⁻)
n = 9
p=0.8238
n = 7
NKT cells (% of CD3⁺)
n = 9
p=0.8017
n = 7
B cells (% of total)
n = 9
p=0.9465
n = 7
NK cells (% of CD3⁻)
n = 6
p=0.0516
n = 5
NKT cells (% of CD3⁺)
n = 7
p=0.1716
n = 5
B cells (% of total)
n = 7
p=0.0951
n = 5
c
CD4⁺ (% of CD3⁺)
n = 14
p=0.9291
n = 7
CD8⁺ (% of CD3⁺)
n = 14
p=0.2493
n = 7
FOXP3⁺ (% of CD4⁺)
n = 14
p=0.6162
n = 6
Ki67⁺ (% of CD4⁺)
n = 13
p=0.0552
n = 7
Ki67⁺ (% of CD8⁺)
n = 13
p=0.1452
n = 7
Ki67⁺ (% of FOXP3⁺)
n = 14
p=0.5794
n = 6
NK cells (% of CD3⁻)
n = 4
p=0.0973
n = 7
NKT cells (% of CD3⁺)
n = 4
p=0.8321
n = 7
B cells (% of total)
n = 4
p<0.0001
n = 7
d
α-CD4 + α-CD8 (n = 14)
α-CD4 + α-CD8 + NAM (n = 14)
Area (cm²)
Days after treatment
OS (%)
HR: 0.92 [0.44;1.94]
p=0.8017
e
α-IFNAR1 (n = 16)
α-IFNAR1 + NAM (n = 14)
HR: 0.91 [0.44;1.85]
p=0.7300
f
α-IFNγ (n = 16)
α-IFNγ + NAM (n = 14)
HR: 0.87 [0.44;1.77]
p=0.6183
g
α-IL17 (n = 8)
α-IL17 + NAM (n = 8)
HR: 0.73 [0.22;2.38]
p=0.5816
h
Control (n = 13)
NAM (n = 15)
MTX (n = 13)
MTX + NAM (n = 16)
p=0.0025, HR: 0.35 [0.15;0.84]
p<0.0001, HR: 0.26 [0.10;0.68]
p<0.0001, HR: 0.26 [0.10;0.66]
Area (cm²)
OS (%)
Days after treatment

Supplementary Figure 4. **Immunostimulatory effects of NAM. a-c.** Relative abundance and proliferative potential (based on Ki67 positivity) of $CD8^+$ T cells, $CD4^+$ T cells, $CD4^+CD25^+FOXP3^+$ T cells, $CD3^-CD56^+$ NK cells, $CD3^+CD56^+$ NKT cells, and $CD19^+B220^+$ B cells in the tumor (**a**), spleen (**b**) and lymph nodes (**c**) of C57BL/6 mice with established M/D-driven tumors that were maintained in control conditions or subjected to NAM supplementation with the drinking water until lesions reached 70-100 mm^2 surface area. Results are means ± SEM and individual data points. Number of mice and p values (unpaired, two-sided Student's t) are reported. **d-g.** Tumor growth and overall survival (OS) in C57BL/6 mice bearing established AT3 tumors that received NAM supplementation with the drinking water alone or along with co-depletion of $CD4^+$ and $CD8^+$ T cells (**d**) or the neutralization of IFNAR1 (**e**), IFNG (**f**) or IL17 (**g**). Tumor growth results are means ± SEM. Number of mice, HR and p values (two-sided log-rank) are reported. **h.** Tumor growth and OS in C57BL/6 mice bearing established M/D-driven tumors that were left untreated or received NAM supplementation with the drinking water, intraperitoneal mitoxantrone (MTX) treatment or both. Number of mice, Tumor growth results are means ± SEM. Number of mice, HR and p values (two-sided log-rank) are reported.

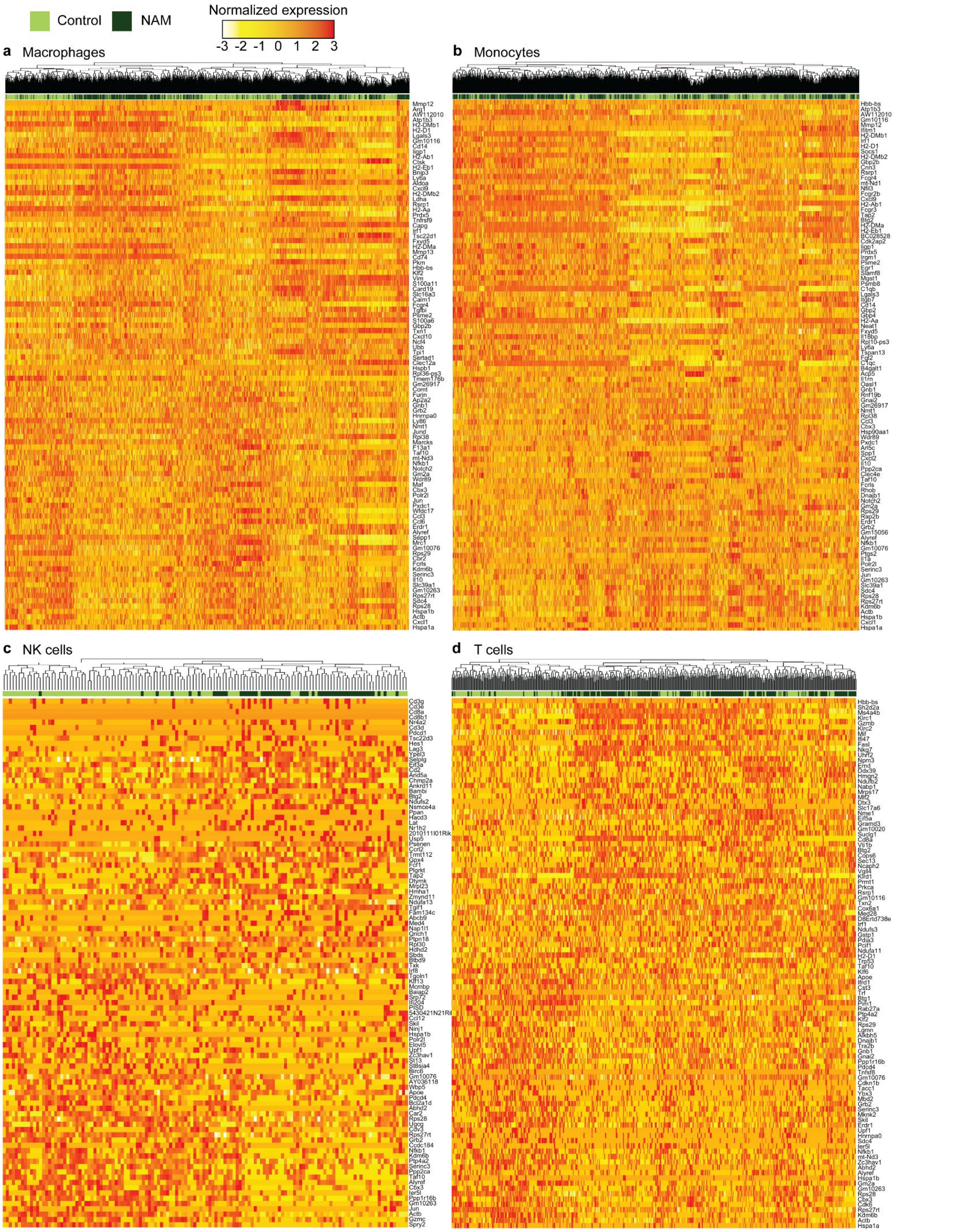

Control
NAM
Normalized expression
-3 -2 -1 0 1 2 3
a Macrophages
Mmp12
Arg1
AW112010
Atp1b3
H2-DMb1
H2-D1
Lgals3
Gm10116
Cd14
Iigp1
H2-Ab1
Ctsk
H2-Eb1
Bnip3
Ly6a
Aldoa
Cxcl9
H2-DMb2
Ldha
Rsrp1
H2-Aa
Prdx5
Tnfrsf9
Capg
Irf1
Tsc22d1
Fxyd5
H2-DMa
Mmp13
Cd74
Pkm
Hbb-bs
Klf2
Vim
S100a11
Card19
Slc16a3
Calm1
Fcgr4
Tgfbi
Psme2
S100a6
Gbp2b
Txn1
Cxcl10
Ncf4
Ubb
Tpi1
Sertad1
Clec12a
Hspb1
Rpl36-ps3
Tmem176b
Gm26917
Comt
Furin
Ap2a2
Gnb1
Grb2
Hnrnpa0
Ly86
Nmt1
Jund
Rpl38
Marcks
F13a1
Taf10
mt-Nd3
Nfkb1
Notch2
Gm2a
Wdr89
Maf
Cbx3
Polr2l
Jun
Pxdc1
Wfdc17
Ccl3
Ccl6
Erdr1
Alyref
Sepp1
Mrc1
Gm10076
Rps29
Cbr2
Fcrls
Kdm6b
Serinc3
Il10
Slc39a1
Gm10263
Rps27rt
Sdc4
Rps28
Hspa1b
Actb
Cxcl1
Hspa1a
b Monocytes
Hbb-bs
Atp1b3
AW112010
Gm10116
Mmp12
Ifitm1
H2-DMb1
Irf1
H2-D1
Socs1
H2-DMb2
Gbp2b
Cnn3
Rsrp1
Fcgr4
mt-Nd1
Nfil3
Fcgr2b
Cxcl9
H2-Ab1
Fcgr3
Tap2
Btg2
H2-DMa
H2-Eb1
BC028528
Cdk2ap2
Iigp1
Prdx5
Irgm1
Psme2
Egr1
Slamf8
Mgst1
Psmb8
C1qb
Lgals3
Itgb7
Cd14
Gbp2
Gbp4
H2-Aa
Neat1
Fxyd5
Il18bp
Rpl10-ps3
Ly6a
Tspan13
Fgl2
C1qc
B4galt1
Acp5
Il1rn
Oasl1
Gnb1
Rnf19b
Gnai2
Gm26917
Nmt1
Rpl38
Ccl3
Cbx3
Hsp90aa1
Wdr89
Pxdc1
Arl5c
Spp1
Cxcl2
Il10
Ppp2ca
Clec4e
Taf10
Fcrls
Rhob
Dnajb1
Notch2
Gm2a
Rps29
Rap2b
Erdr1
Grb2
Gm15056
Alyref
Nfkb1
Gm10076
Ptgs2
Il1a
Polr2l
Serinc3
Jun
Gm10263
Slc39a1
Sdc4
Rps28
Rps27rt
Kdm6b
Actb
Hspa1b
Cxcl1
Hspa1a
c NK cells
Cd3g
Cd3e
Cd8a
Cd8b1
Nr4a2
Cd3d
Pdcd1
Tsc22d3
Hes1
Lag3
Ypel3
Selplg
Eif3a
Cd2
Arid5a
Chmp2a
Ankrd11
Bambi
Btg2
Ndufs2
Nsmce4a
Ppan
Hacd3
Lat
Nr1h2
2010111I01Rik
Usp5
Psenen
Ccrl2
Trmt112
Gpx4
Fcf1
Plgrkt
Tap2
Dtymk
Mrpl23
Hmha1
Zmynd11
Ndufa13
Tgif1
Fam134c
Abcb9
Med4
Nap1l1
Qrich1
Ptpn18
Rpl30
Hdhd2
Sbds
Btbd9
Txk
Irf8
Tgoln1
Klf13
Mcmbp
Baiap2
Srp72
Ifi204
PISD
5430421N21Rik
Ccl12
Skil
Ninj1
Hspa1b
Polr2l
Elovl5
Upf1
Zc3hav1
St13
St8sia4
Birc6
Gm10076
AY036118
Wbp5
Apoe
Pdcd4
Bcl2a1d
Abhd2
Car2
Rps28
Ugcg
Cdv3
Rps27rt
Grb2
Ccdc184
Nfkb1
Kdm6b
Ptp4a2
Serinc3
Ppp2ca
Taf10
Alyref
Cbx3
Ier5l
Ppp1r16b
Gm10263
Jun
Actb
Gzmc
Spry2
d T cells
Hbb-bs
Sh2d2a
Ms4a4b
Klrc1
Gzmb
Klrc2
Mif
Ifi47
Fasl
Nkg7
Uhrf2
Npm3
Emd
Ddx39
Hmgn2
Ndufb2
Nabp1
Mrps17
Mff2
Dtx3
Slc17a6
Nme1
Eif5a
Gramd3
Gm10020
Suclg1
Cd8a
Vti1b
Btg2
Cops6
Sec13
Ncaph2
Vgll4
Klrd1
Prmt1
Prkca
Rsrp1
Gm10116
Txn2
Cox6a1
Med28
D8Ertd738e
Irf1
Ndufs3
Gstp1
Pdia3
Pclf1
Ndufa11
H2-D1
Trp53
Taf10
Klf6
Apoe
Ifrd1
Cst3
Trf
Btg1
Prm1
Rab27a
Ptp4a2
Klf2
Rps29
Lgmn
Alkbh5
Dnajb1
Tra2b
Gnb1
Gnai2
Ppp1r16b
Pdcd4
Tnfsf8
Gm10076
Cdkn1b
Tacc1
Ybx3
Mbd2
Grb2
Serinc3
Mknk2
Skil
Erdr1
Upf1
Hnrnpa0
Sdc4
Ier5l
Nfkb1
mt-Nd3
Zc3hav1
Abhd2
Alyref
Hspa1b
Gm2a
Gm10263
Rps28
Cbx3
Cdk6
Rps27rt
Kdm6b
Actb
Hspa1a

Supplementary Figure 5. **Transcriptional alterations induced by NAM in tumor-infiltrating macrophages, monocytes, T cells and NK cells. a-d.** Unsupervised hierarchical clusters of the top 100 differentially expressed genes in tumor-infiltrating macrophages (**a**), monocytes (**b**), T cells (**c**) and NK cells (**d**) from TSA tumors established in BALB/c mice that (at tumor detection) were either left untreated (control) or subjected to NAM supplementation with the drinking water for 10 consecutive days. Gene names, fold change and unadjusted, one-sided *p* values (based on the MAST model) are available in **Supplementary Data 1**.

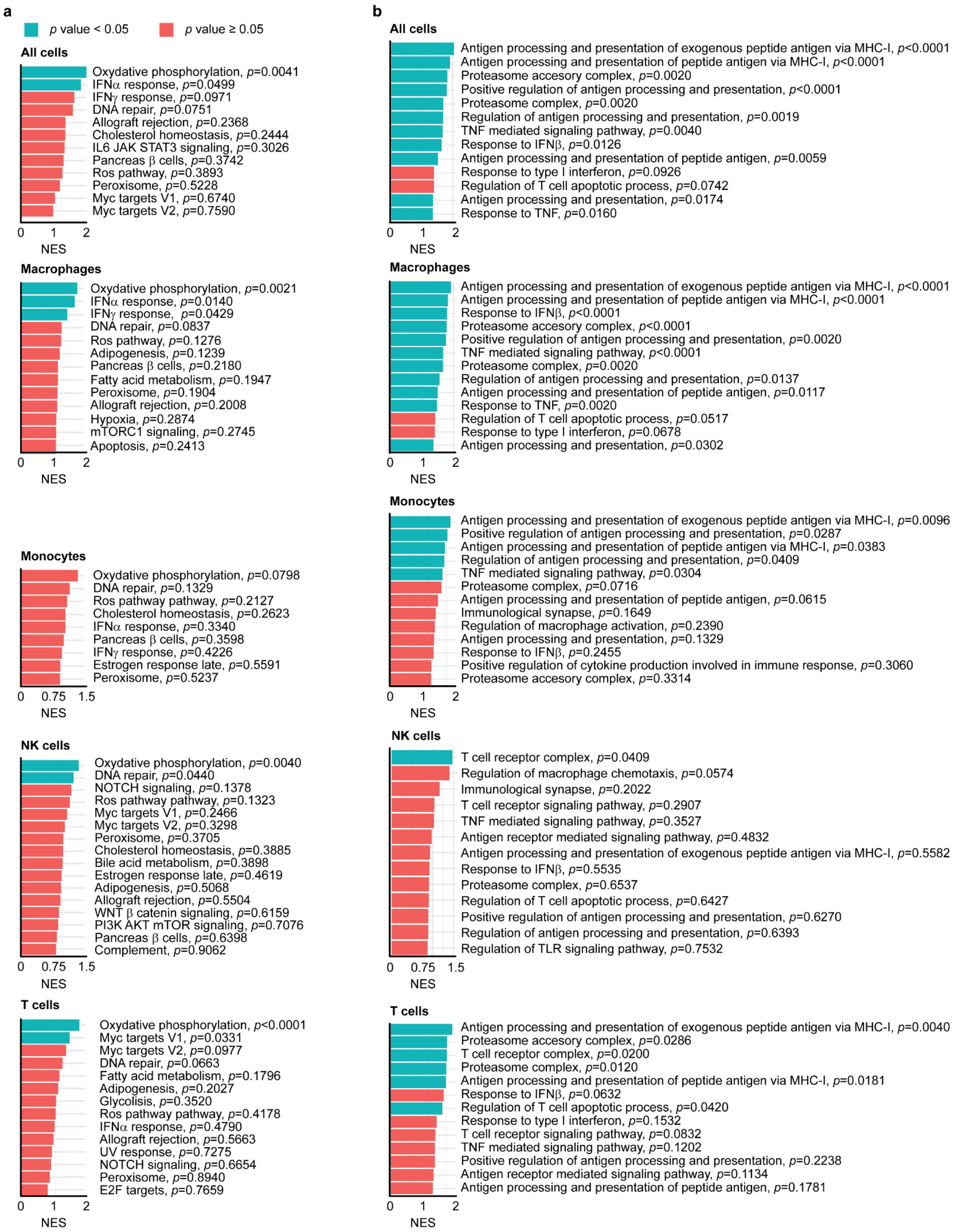
a
p value < 0.05
p value ≥ 0.05
All cells
Oxydative phosphorylation, p=0.0041
IFNα response, p=0.0499
IFNγ response, p=0.0971
DNA repair, p=0.0751
Allograft rejection, p=0.2368
Cholesterol homeostasis, p=0.2444
IL6 JAK STAT3 signaling, p=0.3026
Pancreas β cells, p=0.3742
Ros pathway, p=0.3893
Peroxisome, p=0.5228
Myc targets V1, p=0.6740
Myc targets V2, p=0.7590
0 1 2
NES
Macrophages
Oxydative phosphorylation, p=0.0021
IFNα response, p=0.0140
IFNγ response, p=0.0429
DNA repair, p=0.0837
Ros pathway, p=0.1276
Adipogenesis, p=0.1239
Pancreas β cells, p=0.2180
Fatty acid metabolism, p=0.1947
Peroxisome, p=0.1904
Allograft rejection, p=0.2008
Hypoxia, p=0.2874
mTORC1 signaling, p=0.2745
Apoptosis, p=0.2413
0 1 2
NES
Monocytes
Oxydative phosphorylation, p=0.0798
DNA repair, p=0.1329
Ros pathway pathway, p=0.2127
Cholesterol homeostasis, p=0.2623
IFNα response, p=0.3340
Pancreas β cells, p=0.3598
IFNγ response, p=0.4226
Estrogen response late, p=0.5591
Peroxisome, p=0.5237
0 0.75 1.5
NES
NK cells
Oxydative phosphorylation, p=0.0040
DNA repair, p=0.0440
NOTCH signaling, p=0.1378
Ros pathway pathway, p=0.1323
Myc targets V1, p=0.2466
Myc targets V2, p=0.3298
Peroxisome, p=0.3705
Cholesterol homeostasis, p=0.3885
Bile acid metabolism, p=0.3898
Estrogen response late, p=0.4619
Adipogenesis, p=0.5068
Allograft rejection, p=0.5504
WNT β catenin signaling, p=0.6159
PI3K AKT mTOR signaling, p=0.7076
Pancreas β cells, p=0.6398
Complement, p=0.9062
0 0.75 1.5
NES
T cells
Oxydative phosphorylation, p<0.0001
Myc targets V1, p=0.0331
Myc targets V2, p=0.0977
DNA repair, p=0.0663
Fatty acid metabolism, p=0.1796
Adipogenesis, p=0.2027
Glycolisis, p=0.3520
Ros pathway pathway, p=0.4178
IFNα response, p=0.4790
Allograft rejection, p=0.5663
UV response, p=0.7275
NOTCH signaling, p=0.6654
Peroxisome, p=0.8940
E2F targets, p=0.7659
0 1 2
NES
b
All cells
Antigen processing and presentation of exogenous peptide antigen via MHC-I, p<0.0001
Antigen processing and presentation of peptide antigen via MHC-I, p<0.0001
Proteasome accesory complex, p=0.0020
Positive regulation of antigen processing and presentation, p<0.0001
Proteasome complex, p=0.0020
Regulation of antigen processing and presentation, p=0.0019
TNF mediated signaling pathway, p=0.0040
Response to IFNβ, p=0.0126
Antigen processing and presentation of peptide antigen, p=0.0059
Response to type I interferon, p=0.0926
Regulation of T cell apoptotic process, p=0.0742
Antigen processing and presentation, p=0.0174
Response to TNF, p=0.0160
0 1 2
NES
Macrophages
Antigen processing and presentation of exogenous peptide antigen via MHC-I, p<0.0001
Antigen processing and presentation of peptide antigen via MHC-I, p<0.0001
Response to IFNβ, p<0.0001
Proteasome accesory complex, p<0.0001
Positive regulation of antigen processing and presentation, p=0.0020
TNF mediated signaling pathway, p<0.0001
Proteasome complex, p=0.0020
Regulation of antigen processing and presentation, p=0.0137
Antigen processing and presentation of peptide antigen, p=0.0117
Response to TNF, p=0.0020
Regulation of T cell apoptotic process, p=0.0517
Response to type I interferon, p=0.0678
Antigen processing and presentation, p=0.0302
0 1 2
NES
Monocytes
Antigen processing and presentation of exogenous peptide antigen via MHC-I, p=0.0096
Positive regulation of antigen processing and presentation, p=0.0287
Antigen processing and presentation of peptide antigen via MHC-I, p=0.0383
Regulation of antigen processing and presentation, p=0.0409
TNF mediated signaling pathway, p=0.0304
Proteasome complex, p=0.0716
Antigen processing and presentation of peptide antigen, p=0.0615
Immunological synapse, p=0.1649
Regulation of macrophage activation, p=0.2390
Antigen processing and presentation, p=0.1329
Response to IFNβ, p=0.2455
Positive regulation of cytokine production involved in immune response, p=0.3060
Proteasome accesory complex, p=0.3314
0 1 2
NES
NK cells
T cell receptor complex, p=0.0409
Regulation of macrophage chemotaxis, p=0.0574
Immunological synapse, p=0.2022
T cell receptor signaling pathway, p=0.2907
TNF mediated signaling pathway, p=0.3527
Antigen receptor mediated signaling pathway, p=0.4832
Antigen processing and presentation of exogenous peptide antigen via MHC-I, p=0.5582
Response to IFNβ, p=0.5535
Proteasome complex, p=0.6537
Regulation of T cell apoptotic process, p=0.6427
Positive regulation of antigen processing and presentation, p=0.6270
Regulation of antigen processing and presentation, p=0.6393
Regulation of TLR signaling pathway, p=0.7532
0 0.75 1.5
NES
T cells
Antigen processing and presentation of exogenous peptide antigen via MHC-I, p=0.0040
Proteasome accesory complex, p=0.0286
T cell receptor complex, p=0.0200
Proteasome complex, p=0.0120
Antigen processing and presentation of peptide antigen via MHC-I, p=0.0181
Response to IFNβ, p=0.0632
Regulation of T cell apoptotic process, p=0.0420
Response to type I interferon, p=0.1532
T cell receptor signaling pathway, p=0.0832
TNF mediated signaling pathway, p=0.1202
Positive regulation of antigen processing and presentation, p=0.2238
Antigen receptor mediated signaling pathway, p=0.1134
Antigen processing and presentation of peptide antigen, p=0.1781
0 1 2
NES

Supplementary Figure 6. **NAM-treated tumors exhibit improved antigen presentation and superior cytotoxic functions. a, b.** Gene Ontology (GO, **a**) and Hallmark (**b**) gene set enrichment analysis of differentially expressed genes in the indicated immune cell populations from TSA tumors established in BALB/c mice that were maintained in control conditions or subjected to NAM supplementation with the drinking water for 10 consecutive days after tumor detection. Only GO terms linked to immunity plus all Hallmarks terms were tested, and only those enriched in NAM-treated tumors are reported. Statistical significance was determined by fast gene set enrichment analysis (FGSEA). Unadjusted, two-sided p values for enrichment are reported. NES, normalized enrichment score.

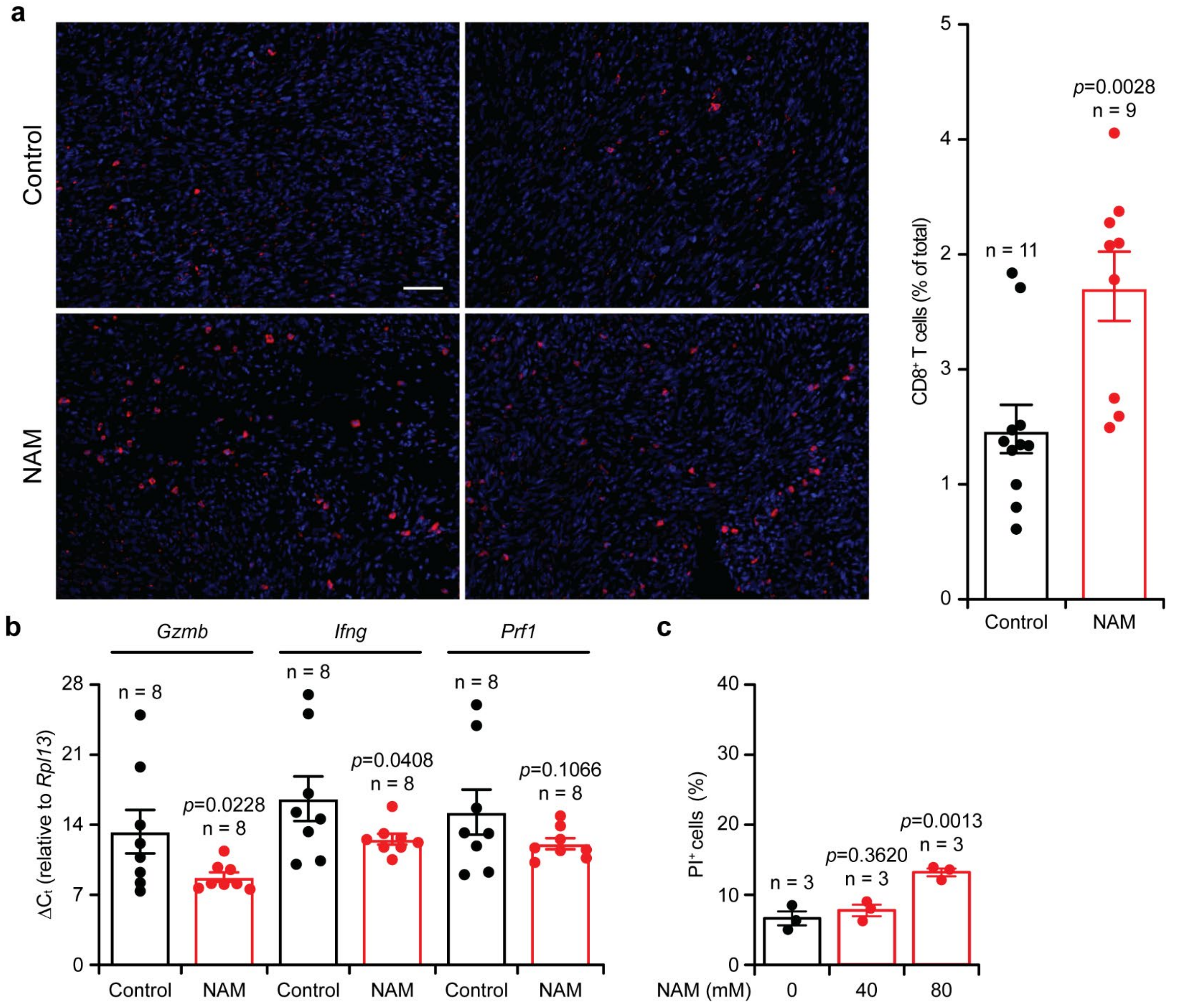

Supplementary Figure 7. **Tumor infiltration by immune effectors driven by NAM. a.** $CD8^+$ T cell infiltration in TSA tumors established in BALB/c mice that were maintained in control conditions or subjected to NAM supplementation with the drinking water for 7 consecutive days upon tumor detection, as assessed by immunofluorescence microscopy with a CD8-specific antibody (red) and automated image analysis. Representative images (scale bar = 100 µm) and quantitative data (means ± SEM and individual data points) are reported. Number of images (collected from 3 independent samples per condition) and *p* values (unpaired, two-sided Student's t) are reported. **b.** Transcriptional levels of *GZMB*, *IFNG* and *PRF1* in M/D-driven tumors established in C57BL/6 mice that were maintained in control conditions of subjected to NAM supplementation in the drinking water for 7 consecutive days upon tumor detection. Please note that results are reported as ΔC_t values as compared to *Rpl13* C_t values for each tumor, because $\Delta\Delta C_t$ values cannot be calculated in the absence of uniform control conditions. In this setting, lower ΔC_t correspond to higher transcript levels. Results are mean ± SEM plus individual data points. Number of biologically independent samples collected over three independent experiments and *p* values are reported (one way-ANOVA plus Fisher LSD, as compared to the same transcript in M/D-driven tumors from untreated mice). **c.** Percentage of dead (PI^+) TSA cells upon culture in control conditions on in the presence of NAM at the indicated concentrations for 48 hours. Results are mean ± SEM plus individual data points. Number of biologically independent samples and *p* values (one way-ANOVA plus Fisher LSD, as compared to untreated cells) are reported.

In vitro - Cell death (Suppl. Fig. 7c)

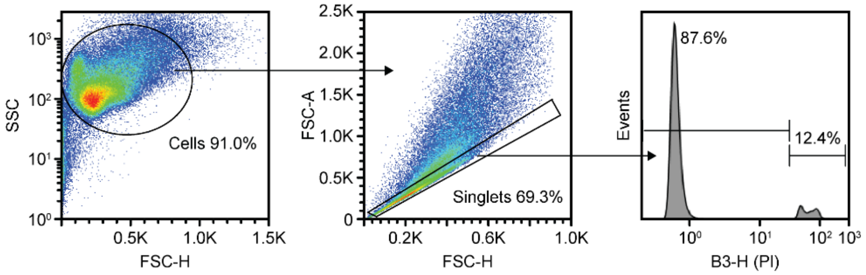

In vitro - NK cell activation (Fig. 8d)

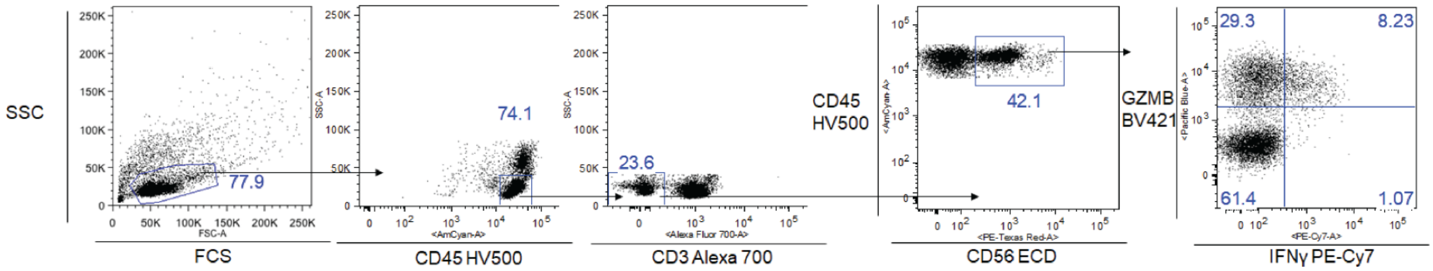

In vitro - T cell activation (Fig. 8c)

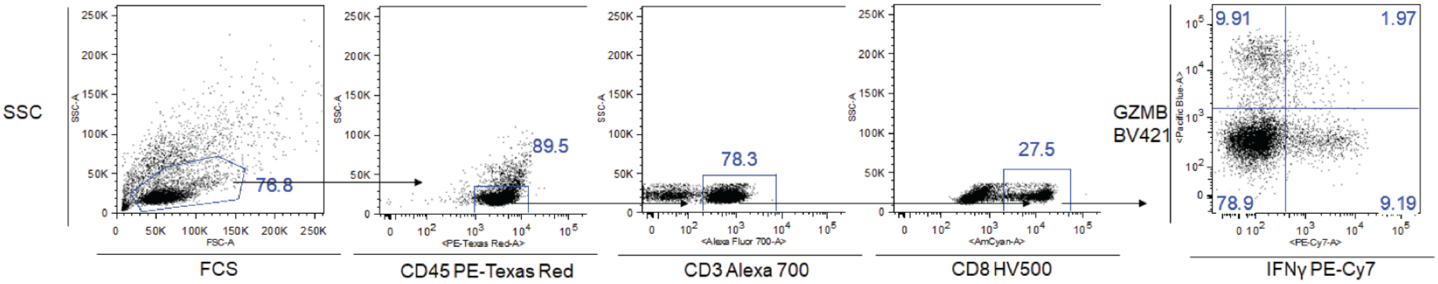

In vivo - Infiltrate phenotyping - Procedure 1 (Fig. 6f)

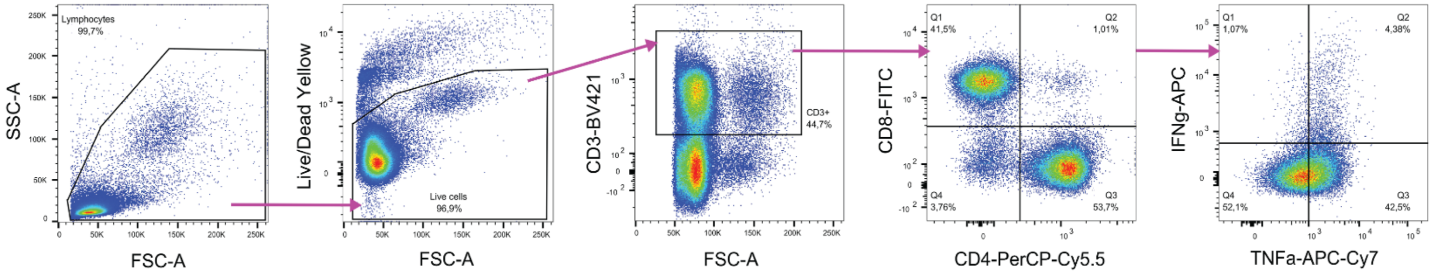

In vivo - Infiltrate phenotyping - Procedure 2
(Fig. 1i; Suppl. Fig. 2d, e; Suppl. Fig. 4a-c)

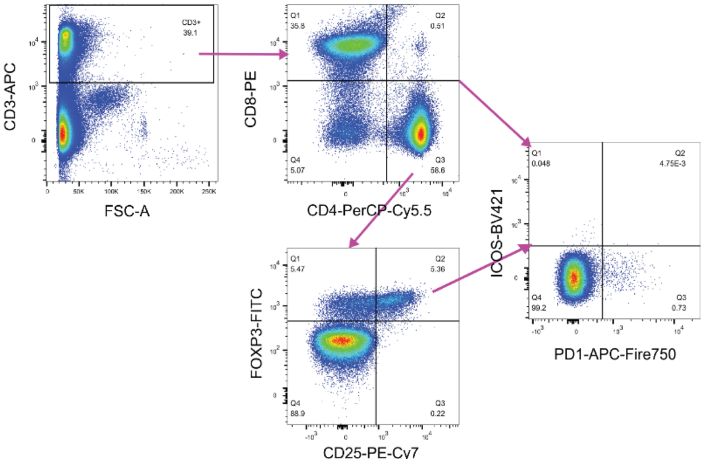

In vivo - Infiltrate phenotyping - Procedure 3
(Suppl. Fig. 2d, e; Suppl. Fig. 4a-c)

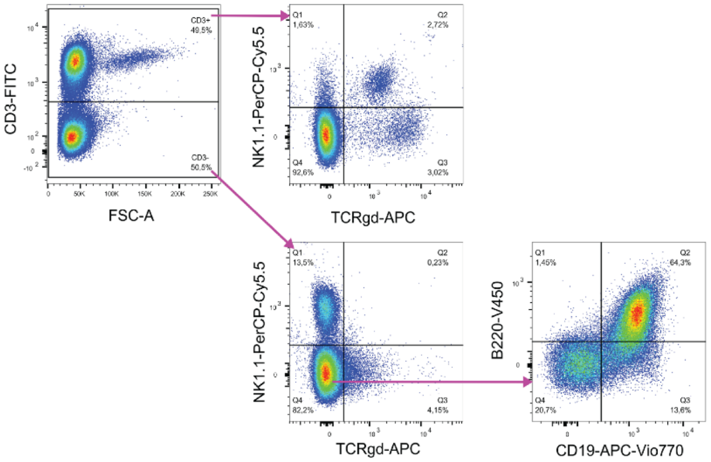

Supplementary Figure 8. **Gating strategies for flow cytometry employed in this study.**
