# Supplementary material for: Immunoprophylactic and immunotherapeutic control of hormone receptor-positive breast cancer
Source: Nat Commun. 2020 Jul 30;11:3819. doi: 10.1038/s41467-020-17644-0 (PMC7393498; doi:10.1038/s41467-020-17644-0)
Supplement: Supplementary file 3 — Description of Additional Supplementary Files [file 41467_2020_17644_MOESM3_ESM.pdf]

Title: Supplementary Data 1.

Description: Top 100 differentially expressed genes in T cells, NK cells, monocytes and macrophages infiltrating control versus NAM-treated TSA tumors. Gene name, log fold change (FC) and unadjusted, one-sided p values (from the MAST model for single-cell gene expression) are reported.
