# Supplementary material for: Immunoprophylactic and immunotherapeutic control of hormone receptor-positive breast cancer
Source: Nat Commun. 2020 Jul 30;11:3819. doi: 10.1038/s41467-020-17644-0 (PMC7393498; doi:10.1038/s41467-020-17644-0)
Supplement: Supplementary file 5 — Reporting Summary [file 41467_2020_17644_MOESM5_ESM.pdf]

nature research

Corresponding author(s): Lorenzo Galluzzi

Last updated by author(s): Jun 30, 2020

Reporting Summary

Nature Research wishes to improve the reproducibility of the work that we publish. This form provides structure for consistency and transparency in reporting. For further information on Nature Research policies, see Authors & Referees and the Editorial Policy Checklist .

Statistics

For all statistical analyses, confirm that the following items are present in the figure legend, table legend, main text, or Methods section.

n/a	Confirmed	
☐	☒	The exact sample size (*n*) for each experimental group/condition, given as a discrete number and unit of measurement
☐	☒	A statement on whether measurements were taken from distinct samples or whether the same sample was measured repeatedly
☐	☒	The statistical test(s) used AND whether they are one- or two-sided<br>*Only common tests should be described solely by name; describe more complex techniques in the Methods section.*
☒	☐	A description of all covariates tested
☐	☒	A description of any assumptions or corrections, such as tests of normality and adjustment for multiple comparisons
☐	☒	A full description of the statistical parameters including central tendency (e.g. means) or other basic estimates (e.g. regression coefficient) AND variation (e.g. standard deviation) or associated estimates of uncertainty (e.g. confidence intervals)
☐	☒	For null hypothesis testing, the test statistic (e.g. *F*, *t*, *r*) with confidence intervals, effect sizes, degrees of freedom and *P* value noted<br>*Give P values as exact values whenever suitable.*
☒	☐	For Bayesian analysis, information on the choice of priors and Markov chain Monte Carlo settings
☒	☐	For hierarchical and complex designs, identification of the appropriate level for tests and full reporting of outcomes
☐	☒	Estimates of effect sizes (e.g. Cohen's *d*, Pearson's *r*), indicating how they were calculated

Our web collection on statistics for biologists contains articles on many of the points above.

Software and code

Policy information about availability of computer code

Data collection

Flow cytometry: LSRFortessa™ Flow Cytometer operated by FACSDiva™ v. 6.2, LSR II Flow Cytometer operated by FACSDiva™ v. 6.1.3, MACSQuant analyzer operated by MACSQuantify™ v. 2.11.

Bulk RNAseq: FastQC v. 0.11.2, Picard-Tools v. 1.119, Samtools v. 1.0, RSeQC v. 2.3.9.

Single-cell RNAseq: HiSeq2500 System operated by embedded Real Time Analysis (RTA) software v. 3.4.4.

Immunofluorescence microscopy: Eclipse TiE Motorized Digital Fluorescence Microscope operated by NIS-Elements AR v. 4.11, Vectra Polaris™ Automated Quantitative Pathology Imaging System operated by Vectra Polaris v.1, Phenochart v. 1.0.8.

RT-PCR: 7500 RT-PCR system operated by embedded software v. 2.3.

NAM quantification: Q Exactive™ Orbitrap Mass Spectrometer coupled to a Vanquish™ HPLC system operated by Xcalibur v. 4.0.27.19.

Immunoblotting: C600 Gel Doc & Western Imaging System operated by cSeries capture v1.6.8.1110 (Azure Biosystem).

Data analysis

Flow cytometry: FlowJo v. 9.5.3 and X.6.2, Prism v. 8.4.

Tumor incidence and growth: TumGrowth v. 1, Prism v. 8.4.

Immunofluorescence microscopy: inForm v. 2.4.6, Prism v. 8.4.

Bulk RNAseq: R computational environment v. 3.2.5, v. 3.4 and v. 3.6.0, Affymetrix© TAC v. 4.0.1, limma v. 3.32.10, DAVID Functional Annotation Tool v. 6.8, genefu v. 2.16.0, ComplexHeatmap v. 2.0.0, STAR v. 2.4.0, Mouse FAST DB v. 2016_1, DESeq2 v. 1.28.1, DAVID

Functional Annotation Tool v. 6.8, Ingenuity® Pathway Analysis v. 52912811.

Single-cell RNAseq: Cell Ranger Single Cell Software suite v2.1.0, proprietary code generated for this study (https://github.com/nyuhuyang/scRNAseq-LorenzoBlood), R computational environment v. 3.5.3, Seurat_2.3.4, harmony v. 0.1.0, SingleR v. 0.2.2, ggpubr v. 0.2, ggplot2 v. 3.1.1.

All other experiments: Excel 2013, GraphPad Prism v. 8.4.

Figure preparation: Illustrator 2020, Photoshop 2020

For manuscripts utilizing custom algorithms or software that are central to the research but not yet described in published literature, software must be made available to editors/reviewers. We strongly encourage code deposition in a community repository (e.g. GitHub). See the Nature Research guidelines for submitting code & software for further information.

Data

Policy information about availability of data

All manuscripts must include a data availability statement. This statement should provide the following information, where applicable:

- Accession codes, unique identifiers, or web links for publicly available datasets
- A list of figures that have associated raw data
- A description of any restrictions on data availability

Transcriptomic data generated in the context of this study have deposited at Gene Expression Omnibus (GEO) and are publicly available under accession numbers GSE150921 (https://www.ncbi.nlm.nih.gov/geo/query/acc.cgi?acc=GSE150921), GSE150966 (https://www.ncbi.nlm.nih.gov/geo/query/acc.cgi?acc=GSE150966), GSE150967 (https://www.ncbi.nlm.nih.gov/geo/query/acc.cgi?acc=GSE150967) and GSE151197 (https://www.ncbi.nlm.nih.gov/geo/query/acc.cgi?acc=GSE151197). The Cancer Genome Atlas (TCGA) breast cancer (BRCA) dataset is publicly available at https://xenabrowser.net/datapages/?cohort=GDC%20TCGA%20Breast%20Cancer%20(BRCA)&removeHub=https%3A%2F%2Fxena.treehouse.gi.ucsc.edu%3A443. The mm10 mouse genome assembly and ENSEMBL Genes 100 can be accessed at https://useast.ensembl.org/Mus_musculus/Info/Index and https://www.ensembl.org/biomart/martview/eb26fab860902330900c3b118e3cd906, respectively. GO terms, KEGG pathways, REACTOME pathways and Hallmarks terms are freely available to academic users at http://geneontology.org/docs/downloads/, https://www.kegg.jp/kegg/download/, https://reactome.org/download-data, and https://www.gsea-msigdb.org/gsea/msigdb/collections.jsp#H, respectively. Uncropped immunoblotting membranes for Supplementary Fig. 2a, 3e and 3f are provided as a Source Data file with this paper. Additional data supporting the findings of this study are available from the corresponding authors upon reasonable request. A reporting summary for this article is available as a Supplementary Information File.

Field-specific reporting

Please select the one below that is the best fit for your research. If you are not sure, read the appropriate sections before making your selection.

[x] Life sciences [ ] Behavioural & social sciences [ ] Ecological, evolutionary & environmental sciences

For a reference copy of the document with all sections, see nature.com/documents/nr-reporting-summary-flat.pdf

Life sciences study design

All studies must disclose on these points even when the disclosure is negative.

Sample size	For prophylactic and therapeutic studies in mice, groups of at least 10 animals were determined to have 80% power to detect effect size as small as 1 at a two-sided 0.05 significance. Whenever possible, larger groups were used to improve statistical power. For in vitro experiments: no statistical methods were used to determine sample size. A minimum of three biologically independent samples were tested based on previous studies, and experiments were conducted in at least 2 independent instances with similar results. When this turned out to be insufficient to clarify statistically sub-significant trends between groups, sample number was increased to improve statistical power.
Data exclusions	Mice succumbing of tumor-unrelated causes (<1% of mice included in this study), as determined by autopsy upon euthanasia imposed by distress symptoms, were removed from the analysis (predetermined criterion applied to all experiments). Individual data points from in vitro experiments were removed from the analysis when failing initial quality checks (e.g., bacterial contamination, insufficient number of cells), a predetermined criterion applied to all experiments.
Replication	All experiments were conducted in at least 2 independent instances (mostly 3 times) with similar results unless otherwise noted in the Legends of Specific Figures. In each individual experiment, each technical replicate was measured once. All repeats performed showed similar trends. Transcriptomic and scRNAseq findings are from a single experiment involving the indicated number of conditions.
Randomization	Mice were randomly allocated to treatment group at tumor detection. Randomization was not relevant for in vitro studies as they involved cancer cell lines or primary cells from the same origin. Potential covariates such as culture conditions were kept constant throughout the duration of the study. Control conditions from independent experiments performed apart in time were systematically ensure the absence of confounders.
Blinding	Blinding was not implemented in this study. In vivo, in vitro and in silico data were analyzed by software with objective outcomes, and hence blinding was not relevant for the study. Non-blinded confirmation by independent operators was implemented for TFS assessments.

Reporting for specific materials, systems and methods

We require information from authors about some types of materials, experimental systems and methods used in many studies. Here, indicate whether each material, system or method listed is relevant to your study. If you are not sure if a list item applies to your research, read the appropriate section before selecting a response.

Materials & experimental systems

n/a	Involved in the study
☐	☒ Antibodies
☐	☒ Eukaryotic cell lines
☒	☐ Palaeontology
☐	☒ Animals and other organisms
☐	☒ Human research participants
☒	☐ Clinical data

Methods

n/a	Involved in the study
☒	☐ ChIP-seq
☐	☒ Flow cytometry
☒	☐ MRI-based neuroimaging

Antibodies

Antibodies used

Antibodies

anti-CD4 (clone GK1.5, #BE0003-1, Bioxcell™)
https://bxcell.com/product/m-cd4/

anti-CD8alpha(clone 2.43, #BE0061, Bioxcell™)
https://bxcell.com/product/m-cd8a-2/

anti-NKG2D (clone HMG2D, #BE0111, Bioxcell™)
https://bxcell.com/product/m-nkg2d/

anti-NK1.1 (clone PK136, #BE0036, Bioxcell™)
https://bxcell.com/product/nk-1-1/

anti-PD-1 (clone RMP1-14, #BE0146, Bioxcell™)
https://bxcell.com/product/invivomab-anti-m-pd-1/

anti-IFNγ (clone R4-6A2, #BE0054, Bioxcell™)
https://bxcell.com/product/m-inf-gamma/

anti-IFNAR-1 (clone MAR1-5A3, #BE0241, Bioxcell™)
https://bxcell.com/product/anti-m-ifnar-1/

anti-IL17A (clone 17F3, #BE0173, Bioxcell™)
https://bxcell.com/product/m-il-17a/

anti-Annexin A1 (ANXA1) (Clone 29, #610067, BD Biosciences™)
https://www.bdbiosciences.com/ds/pm/tds/610067.pdf, in vivo validated by Vacchelli E. et al., Science, 2015

anti-ESR1-AlexaFluor488 (Clone D-12, #sc-8005, Santa Cruz Biotechnology) https://datasheets.scbt.com/sc-8005.pdf

anti-VIM (polyclonal, #GTX100619, GeneTex™) https://www.genetex.com/PDF/Download?catno=GTX100619

Goat Anti-Rabbit IgG H&L Alexa Fluor® 594 (preadsorbed, #ab150084, Abcam) https://www.abcam.com/goat-rabbit-igg-hl-alexa-fluor-594-preadsorbed-ab150084.pdf

Anti-CD8a Monoclonal Antibody (Clone 4SM15, #14-0808-80, Thermo Fisher™)
https://www.thermofisher.com/order/genome-database/generatePdf?productName=CD8a&assayType=PRANT&detailed=true&productId=14-0808-80

ImmPRESSTM HRP anti-rat IgG (#MP-7444-15, Vector Laboratories™)
https://vectorlabs.com/productpdf/download/file/id/1147/name/ImmPRESS%25C2%25AE_HRP_Goat_Anti-Rat_IgG%252C_Mouse_adsorbed_Polymer_Detection_Kit%252C_Peroxidase.pdf/

anti-LC3B (Polyclonal, #2775, Cell Signaling Technology)

https://www.cellsignal.com/datasheet.jsp?productId=2775&images=1&protocol=0

anti-SQSTM1 (Polyclonal, #5114, Cell Signaling Technology)
https://www.cellsignal.com/datasheet.jsp?productId=5114&images=1&protocol=0

Anti-Atg7 (Clone ATG7-13, #SAB4200304, Sigma-Aldrich)
https://www.sigmaaldrich.com/content/dam/sigma-aldrich/docs/Sigma/Datasheet/9/sab4200304dat.pdf

anti-β-Actin (Clone 8H10D10, #3700, Cell Signaling)
https://www.cellsignal.com/datasheet.jsp?productId=3700&images=1&protocol=0

Amersham ECL Sheep anti-Mouse IgG, HRP-linked whole Ab dilution (NA931-1ML, GE Healthcare Life Sciences)
https://www.sigmaaldrich.com/catalog/product/sigma/gena9311ml?lang=en&region=US

Amersham ECL Donkey anti-Rabbit IgG, HRP-linked whole Ab HRP (NA934-1ML, GE Healthcare Life Sciences) dilution 1:5000
https://www.sigmaaldrich.com/catalog/product/sigma/gena9341ml?lang=en&region=US

anti-CD45 PE Texas Red (Cone HI30, #MHCD4517, Life Technologies™)
https://www.thermofisher.com/antibody/product/CD45-Antibody-clone-HI30-Monoclonal/MHCD4517#pdp-manuals

anti-CD3 Alexa 700 (Clone MEM-57, #11-261-C100, Exbio™)
https://www.exbio.cz/getattachment/57f1f7b8-cbfe-499e-b06e-ad481026e4c4/A7-202-T100_TDS_-20200615-222517987.pdf.aspx

anti-CD8 HV500 (Clone RPA-T8, #560775, BD Biosciences™)
https://www.bdbiosciences.com/ds/pm/tds/560775.pdf

anti-CD45 V500 (Clone HI30, #560777, BD Bioscience™)
https://www.bdbiosciences.com/ds/pm/tds/560777.pdf

anti-CD3-AlexaFluor700 (Clone OKT3, # A7-631-T100 from EXBIO)
https://www.exbio.cz/getattachment/601c2be6-635a-4b41-95fa-d198780aba88/A7-631-T100_TDS_-20200615-222531367.pdf.aspx

anti-CD56 ECD (Clone N901, #B49214, Beckman Coulter™)
https://www.beckman.com/reagents/coulter-flow-cytometry/antibodies-and-kits/single-color-antibodies/cd56/a82943

anti-IFNγ PE-Cy7 (Clone 4S.B3, #25-7319-82, eBioscience™)
https://www.thermofisher.com/order/genome-database/dataSheetPdf?producttype=antibody&productsubtype=antibody_primary&productId=25-7319-82&version=105

anti-Granzyme B BV421 (Clone GB11, #563389, BD Bioscience™)
https://www.bdbiosciences.com/ds/pm/tds/563389.pdf

anti-mouse CD16/CD32 reagent Mouse BD Fc Block™ (clone 2.4G2, #553141 from BD Biosciences)
https://www.bdbiosciences.com/us/applications/research/b-cell-research/surface-markers/mouse/purified-rat-anti-mouse-cd16cd32-mouse-bd-fc-block-24g2/p/553141

anti-CD3 BV421 (clone 145-2C11, #562600, BD Biosciences™)
https://www.bdbiosciences.com/ds/pm/tds/562600.pdf

anti-CD8 FITC (Clone 53-6.7, #553030, BD Biosciences™)
https://www.bdbiosciences.com/ds/pm/tds/553031.pdf

anti-CD4 PerCP-Cy5.5 (Clone RM4-5, #45-0042-82, Thermo Fisher Scientific™)
https://www.fishersci.com/shop/products/anti-cd4-percp-cy-5-5-clone-rm4-5-bd-0-1mg-percp-cy-5-5/bdb550954#?keyword=true

anti-IFN-γ APC (Clone XMG1.2, #554413, BD Biosciences™)
https://www.bdbiosciences.com/ds/pm/tds/554413.pdf

anti-TNF-α APC-Cy7 (Clone MP6-XT22, #506307, BioLegend™)
https://www.biolegend.com/en-us/global-elements/pdf-popup/apc-cyanine7-anti-mouse-tnf-alpha-antibody-12117?filename=APCCyanine7 anti-mouse TNF-alpha Antibody.pdf&pdfgen=true

anti-IL-2 PE (Clone JES6-%H4, #554428, BD Biosciences™)

https://www.bdbiosciences.com/ds/pm/tds/554428.pdf

anti-CD3 APC (Clone 17A2, #17-0032-82, Thermo Fisher Scientific™)
https://www.thermofisher.com/order/genome-database/dataSheetPdf?producttype=antibody&productsubtype=antibody_primary&productId=17-0032-82&version=105

anti-CD8 PE (Clone 53-6.7, #553032, BD Biosciences™)
https://www.bdbiosciences.com/ds/pm/tds/560775.pdf

anti-CD25 PE-Cy7 (Clone PC61.5, #25-0251-82, Thermo Fisher Scientific™)
https://www.thermofisher.com/order/genome-database/dataSheetPdf?producttype=antibody&productsubtype=antibody_primary&productId=25-0251-82&version=105

anti-PD-1 APC-Fire750 (Clone 29F.1A12, #135239, BioLegend™)
https://www.biolegend.com/en-us/global-elements/pdf-popup/apc-fire-750-anti-mouse-cd279-pd-1-antibody-13637?filename=APCFiretrade 750 anti-mouse CD279 PD-1 Antibody.pdf&pdfgen=true

anti-FoxP3 FITC (Clone FJK-16s, #11-5773-82, Thermo Fisher Scientific™)
https://www.thermofisher.com/order/genome-database/dataSheetPdf?producttype=antibody&productsubtype=antibody_primary&productId=11-5773-82&version=105

anti-Ki67 Alexa Fluor 700 (Clone B56, #561277, BD BiosciencesTM)
https://www.bdbiosciences.com/ds/pm/tds/561277.pdf

anti-CD3 FITC (Clone 17A2, #11-0032-82, Thermo Fisher Scientific™)
https://www.thermofisher.com/order/genome-database/dataSheetPdf?producttype=antibody&productsubtype=antibody_primary&productId=11-0032-82&version=105

anti-NK1.1 PerCP-Cy5.5 (Clone PK136, #551114, BD Bioscience™)
https://www.bdbiosciences.com/ds/pm/tds/561111.pdf

anti-B220-V450 (Clone RA3-6B2, #560473, BD Biosciences™)
https://www.bdbiosciences.com/ds/pm/tds/560473.pdf

anti-CD19 APC-Vio770 (Clone REA749, #130-111-886, Miltenyi Biotec™)
https://www.miltenyibiotec.com/_Resources/Persistent/96d54d505cc6987139eeb7b4aaf00a3ad7c7f880/DS_CD19_Antibody_anti-mouse_APC-Vio%C2%AE_770_REAfinity%E2%84%A2_REA749_130-111-886.pdf

All antibodies were employed from multiple lots.

Validation

Validation was provided by the supplier, all antibodies were commercial and previously employed with success in multiple other studies. Link to TDS has been provided above. Please follow the link or go to the manufacturer website to find all relevant information, testing and references.

Eukaryotic cell lines

Policy information about cell lines

Cell line source(s)

AT3 (#SCC178), TSA (#SCC177) and MCA205 (#SCC173) cells were obtained by Millipore. Cell lines from M/D-driven tumors were generated in house.

Authentication

AT3 and TSA cells were authenticated by short-tandem repeat (STR) profiling. MCA205 cells were used shortly after receipt from the vendor and hence were not authenticated. Cell lines generated in house from M/D-driven tumors were stored in liquid nitrogen shortly after establishment, and invariably employed within 3-10 passages upon thawing. Authentication on cell lines from M/D-driven tumors was not possible as cell lines were derived from endogenous tumors and hence no reference is available for STR profiling.

Mycoplasma contamination

All cell lines were routinely confirmed to be Mycoplasma-free by PCR.

Commonly misidentified lines
(See ICLAC register)

No commonly misidentified cell lines were used in this study.

Animals and other organisms

Policy information about studies involving animals; ARRIVE guidelines recommended for reporting animal research

Laboratory animals

WT or genetically modified C57BL/6 and BALB/c female mice (Mus musculus) of 6-15 weeks of age were employed. Mice were maintained in specific pathogen-free standard housing conditions (20+/-2 C, 50+/-5 % humidity, 12h-12h light-dark cycles, food

	and water ad libitum, unless otherwise specified as per experimental design).
Wild animals	No wild animals were used in this study
Field-collected samples	No field-collected samples were used in this study
Ethics oversight	Animal experiments followed the Federation of European Laboratory Animal Science Association (FELASA) guidelines, were in compliance with the EU Directive 63/2010 (protocol 2012_034A) and were approved by institutional ethical committees for animal experimentation at Gustave Roussy (n° 2016031417225217), Centre de Recherche des Cordeliers (n° 2016041518388910), and Weill Cornell Medical College (n° 2017-0007 and 2018-0002).

Note that full information on the approval of the study protocol must also be provided in the manuscript.

Human research participants

Policy information about studies involving human research participants

Population characteristics	Six healthy volunteers, aged 30-35 years old, sex ratio 1:1, no ongoing disoders with symptomatic manifestations, normal blood counts.
Recruitment	Participants (healthy volunteers) were recruited on a public call at University Hospital Motol, Czech Republic. The study design was not prone to self-selection bias as human participants donated PBMCs that were tested for responsiveness to stimulation in a quantitative manner. Participants were checked for general health status on the day of enrollment to avoid potential illnesses that may have confounded the study results.
Ethics oversight	University Hospital Motol, Czech Republic

Note that full information on the approval of the study protocol must also be provided in the manuscript.

Flow Cytometry

Plots

Confirm that:

- [x] The axis labels state the marker and fluorochrome used (e.g. CD4-FITC).
- [x] The axis scales are clearly visible. Include numbers along axes only for bottom left plot of group (a 'group' is an analysis of identical markers).
- [x] All plots are contour plots with outliers or pseudocolor plots.
- [x] A numerical value for number of cells or percentage (with statistics) is provided.

Methodology

Sample preparation	Tumors and mammary glands were recovered, cut with scissors and digested in RPMI medium plus 26.67 µg mL-1 liberase (Sigma-Aldrich) and 0.0167 MU mL-1 DNase I (Sigma-Aldrich) for 30 min at 37°C. RPMI supplemented with 10% FBS was then added to stop enzymes activity, and tumors were crushed on a 100 µm cell strainer with the back of a syringe plunger. After washing, cells were pelleted for 5 min at 300 g and resuspended in 10 mL RPMI medium supplemented with 10% FBS.<br>Spleens and inguinal lymph nodes were collected and crushed between two microscope glass slides. Cells were resuspended in RPMI medium, filtered through 70 µM MACS® SmartStrainers (Miltenyi Biotec™) and pelleted for 5 min at 300 g. Splenocytes and lymph node cells were finally resuspended in 2 mL and 500 µL of RPMI supplemented with 10% FBS, respectively.<br>Peripheral blood mononuclear cells (PBMCs) were isolated from a 10 mL blood aliquot from healthy donors by Ficoll-Paque PLUS (GE Healthcare) gradient centrifugation. PBMCs were then cultured overnight in the presence of 50 ng/mL phorbol 12-myristate 13-acetate (PMA, Calbiochem) plus 1 µg/mL ionomycin alone or combined with NAM, followed by 3 h incubation with brefeldin A (BioLegend).
Instrument	Flow cytometry data were acquired on an LSR II analyzer (BD), a LSR Fortessa analyzer (BD), or MACSQuant 10 (Miltenyi).
Software	Flow cytometry data were acquired with the FACSDiva™ software (BD) or MACSquant v.10 (Miltenyi), and analyzed with FlowJo software (TreeStar, Inc.).
Cell population abundance	No sorting was performed.
Gating strategy	Gating was performed as follows. Invariably, threshold for positivity on one specific channel was determined in control samples stained with the entire antibody/fluorochrome cocktail minus the antibody/fluorochrome emitting in such channel.<br><br>Cell death: cells were identified on SSC/FSC-H dotplots, followed by identification of singlets on FSC-A/FSC-H dotplots, which were analyzed for PI positivity as a single channel (histogram)<br><br>In vitro NK cell activation: cells were identified on SSC/FSC-H dotplots, followed by the identification of CD45+ PMBCs on SSC-CD45 dotplots, CD3-CD45+ cells on SSC/CD3 dotplots, CD3-CD56+ NK cells on CD45/CD56 dotplots, which were assessed for IFNG and GZMB positivity on GZMB/IFNG dotplots.

T cell activation: cells were identified on SSC/FSC-H dotplots, followed by the identification of CD45+ PMBCs on SSC-CD45 dotplots, CD3+CD45+ cells on SSC/CD3 dotplots, CD8+ T cells on SSC/CD8 dotplots, which were assessed for IFNG and GZMB positivity on GZMB/IFNG dotplots.

Infiltrate phenotyping procedure 1: cells were identified based SSC-A/FSC-A dotplots, followed by the identification of live cells based on VividYellow/FSC-A plots, CD3+ cells on CD3/FSC-A plots, CD8+CD4- and CD8-CD4+ cells on CD8/CD4 plots, which were assessed for IFNG, TNFa positivity. IFNG+CD8+CD4- and IFNG+CD4-CD8+ cells were further assessed for IL2 positivity on a TNF/IL2 dotplot.

Infiltrate phenotyping procedure 2: cells were gated as in procedure 1 to identify CD8+CD4- and CD8-CD4+ cells on CD8/CD4 plots, which were assessed as follows: CD8+CD4- and CD8-CD4+ for PD1 and Ki67 positivity on a PD1/Ki67 dotplot; CD8-CD3+ cells for double FOXP3 and CD25 positivity on FOXP3/CD25 dotplots (Tregs), which were assessed for PD1 and Ki67 positivity on a PD1/Ki67 dotplot.

Infiltrate phenotyping procedure 3: cells were gated as in procedure 1 to identify CD3+ and CD3- cells on CD3/FSC-A plots, followed by: identification of NKT cells from the CD3+ gate, based on NK1.1 positivity on a CD3/NK1.1 dotplot, identification of NK cells from the CD3- gate, based on NK1.1 positivity on a CD3/NK1.1 dotplot, and identification of B cells from the CD3- gate, based on double positivity on a B220/CD19 dotplot.

☒ Tick this box to confirm that a figure exemplifying the gating strategy is provided in the Supplementary Information.
