# Supplementary material for: Immunoprophylactic and immunotherapeutic control of hormone receptor-positive breast cancer
Source: Nat Commun. 2020 Jul 30;11:3819. doi: 10.1038/s41467-020-17644-0 (PMC7393498; doi:10.1038/s41467-020-17644-0)
Supplement: Supplementary file 6 — Source Data [file 41467_2020_17644_MOESM6_ESM.zip › Source Data/Suppl. Fig. 2 - Summary.pptx]

## Slide 1
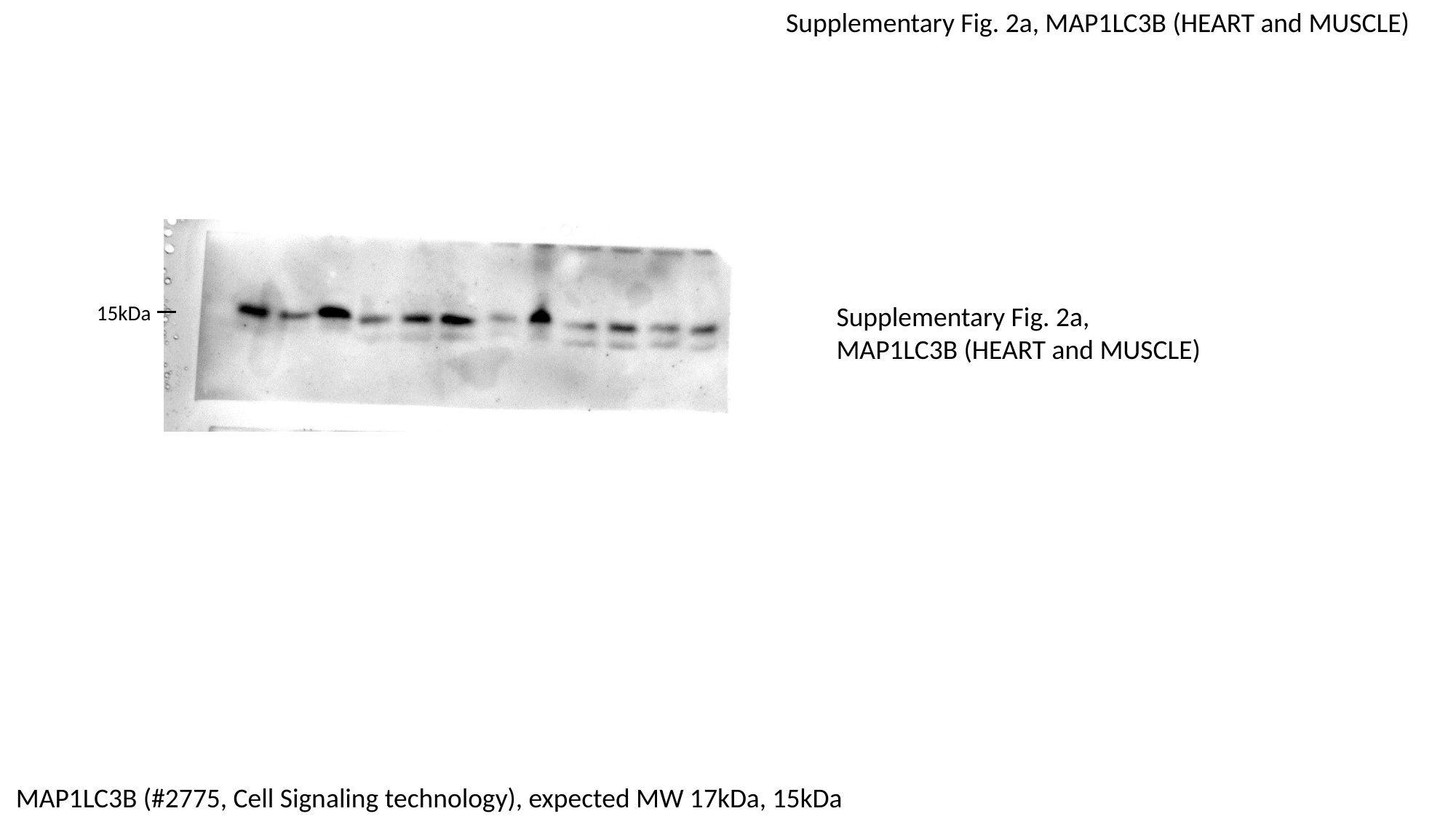

Supplementary Fig. 2a, MAP1LC3B (HEART and MUSCLE)
15kDa
Supplementary Fig. 2a,
MAP1LC3B (HEART and MUSCLE)
MAP1LC3B (#2775, Cell Signaling technology), expected MW 17kDa, 15kDa

## Slide 2
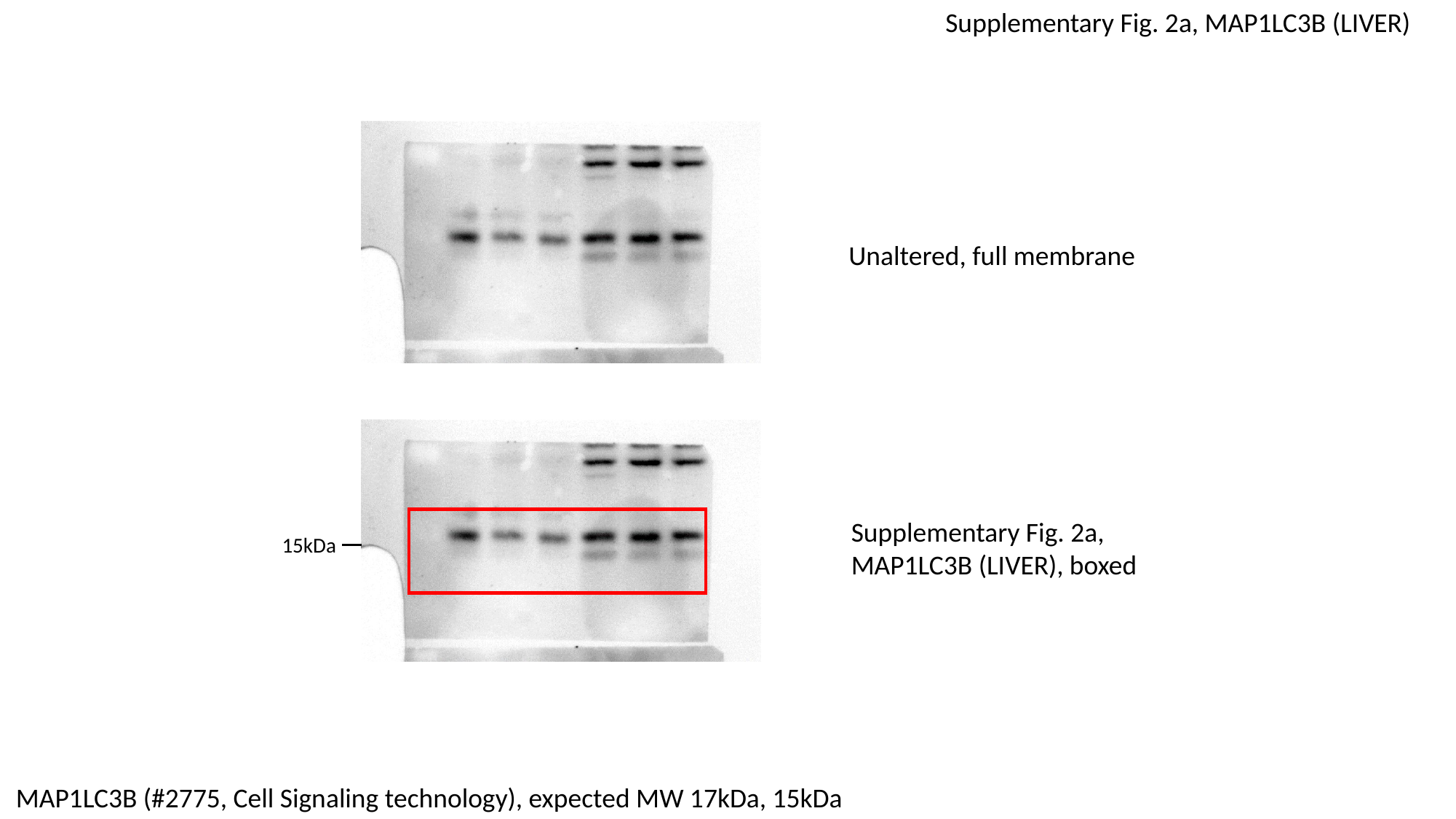

Supplementary Fig. 2a, MAP1LC3B (LIVER)
Unaltered, full membrane
Supplementary Fig. 2a,
MAP1LC3B (LIVER), boxed
15kDa
MAP1LC3B (#2775, Cell Signaling technology), expected MW 17kDa, 15kDa

## Slide 3
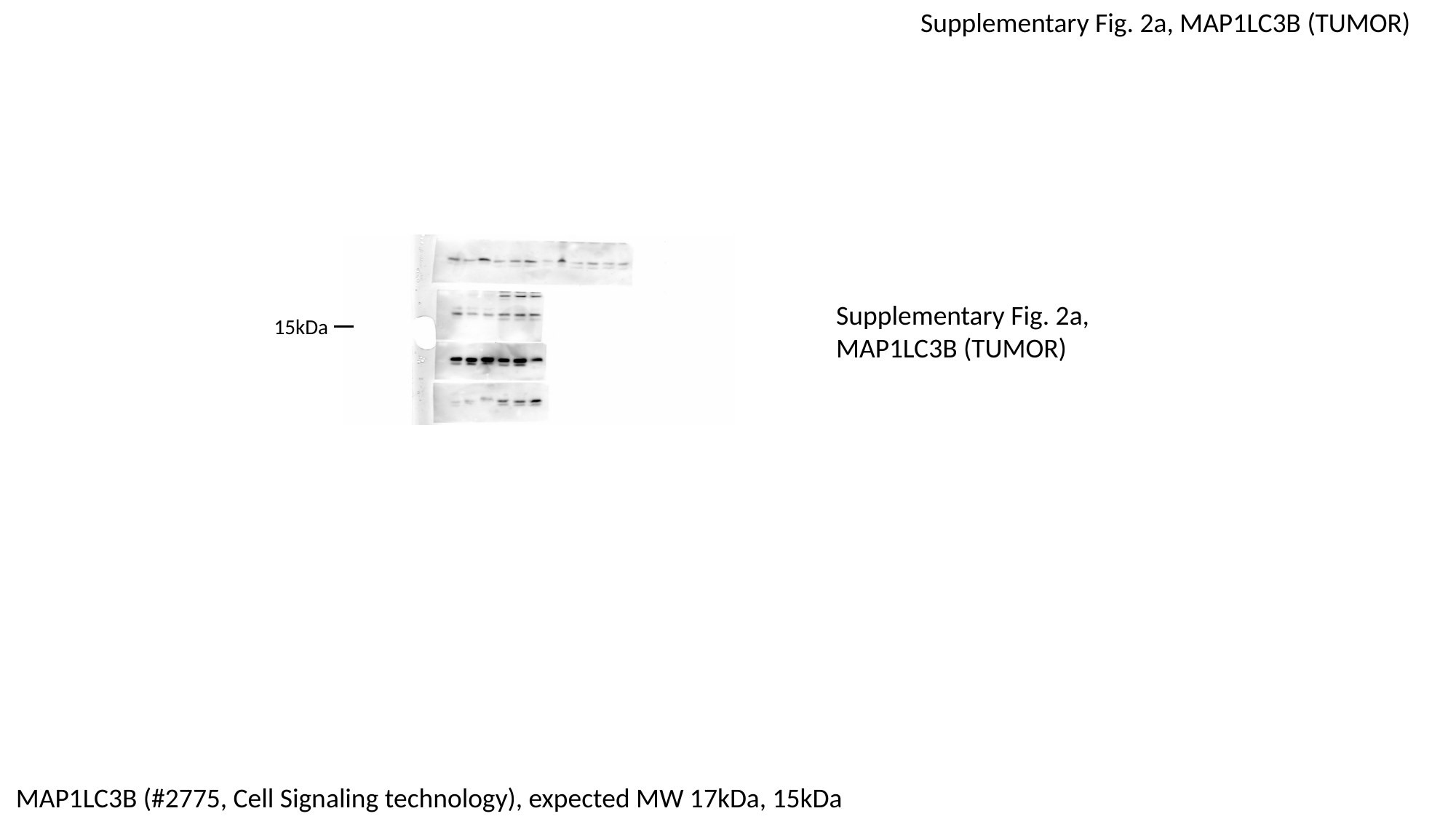

Supplementary Fig. 2a, MAP1LC3B (TUMOR)
Supplementary Fig. 2a,
MAP1LC3B (TUMOR)
15kDa
MAP1LC3B (#2775, Cell Signaling technology), expected MW 17kDa, 15kDa

## Slide 4
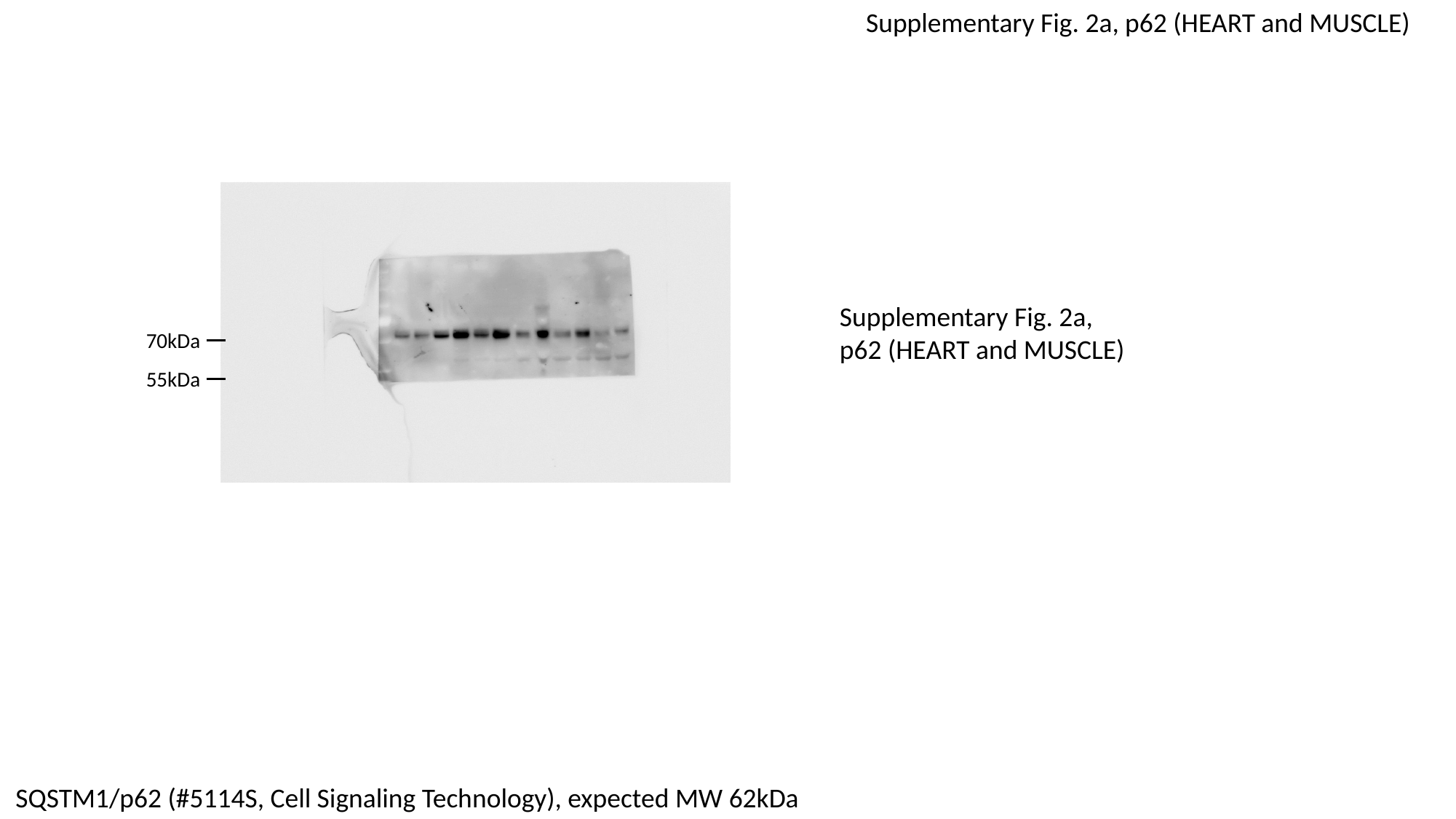

Supplementary Fig. 2a, p62 (HEART and MUSCLE)
Supplementary Fig. 2a,
p62 (HEART and MUSCLE)
70kDa
55kDa
SQSTM1/p62 (#5114S, Cell Signaling Technology), expected MW 62kDa

## Slide 5
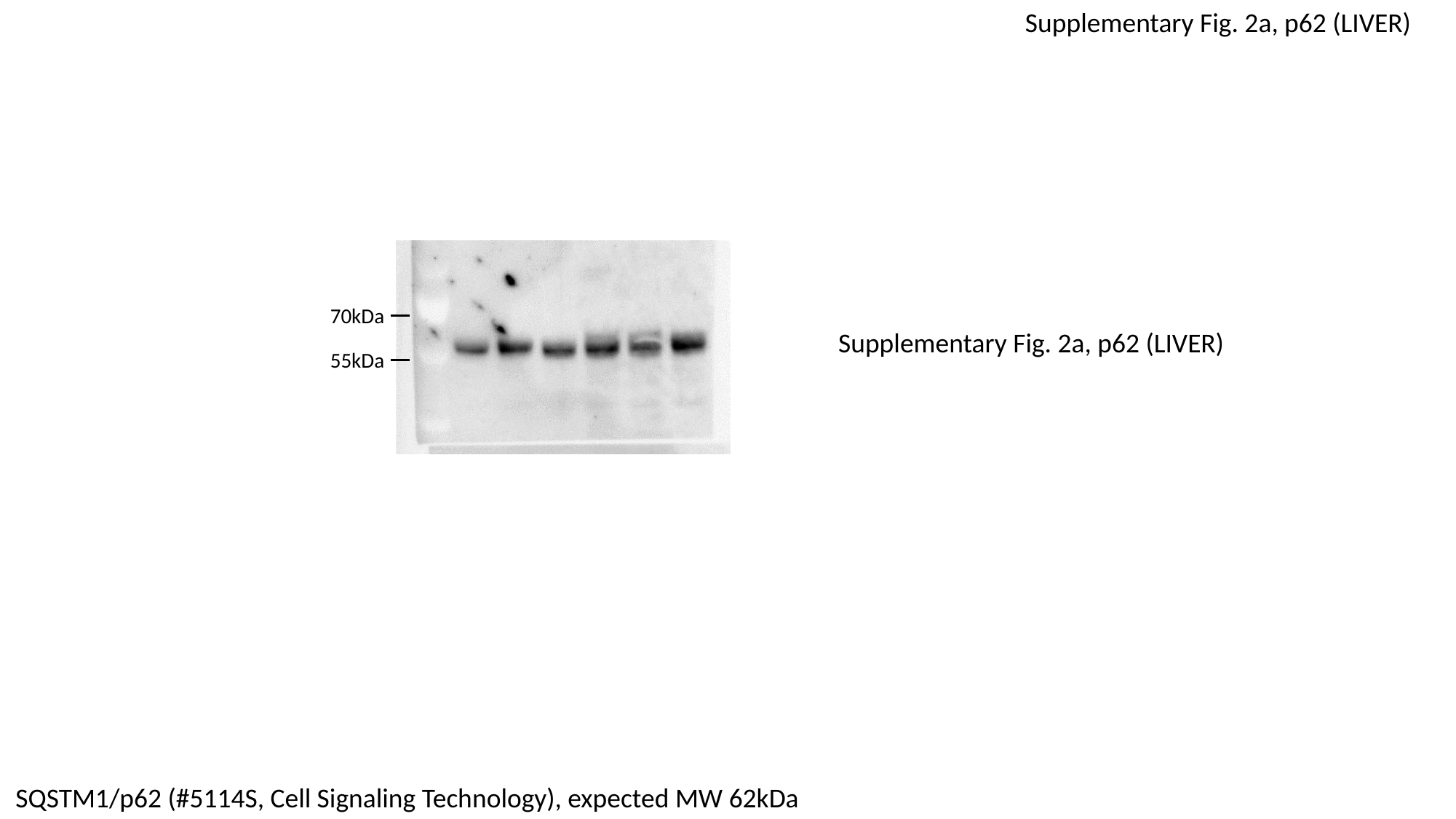

Supplementary Fig. 2a, p62 (LIVER)
70kDa
Supplementary Fig. 2a, p62 (LIVER)
55kDa
SQSTM1/p62 (#5114S, Cell Signaling Technology), expected MW 62kDa

## Slide 6
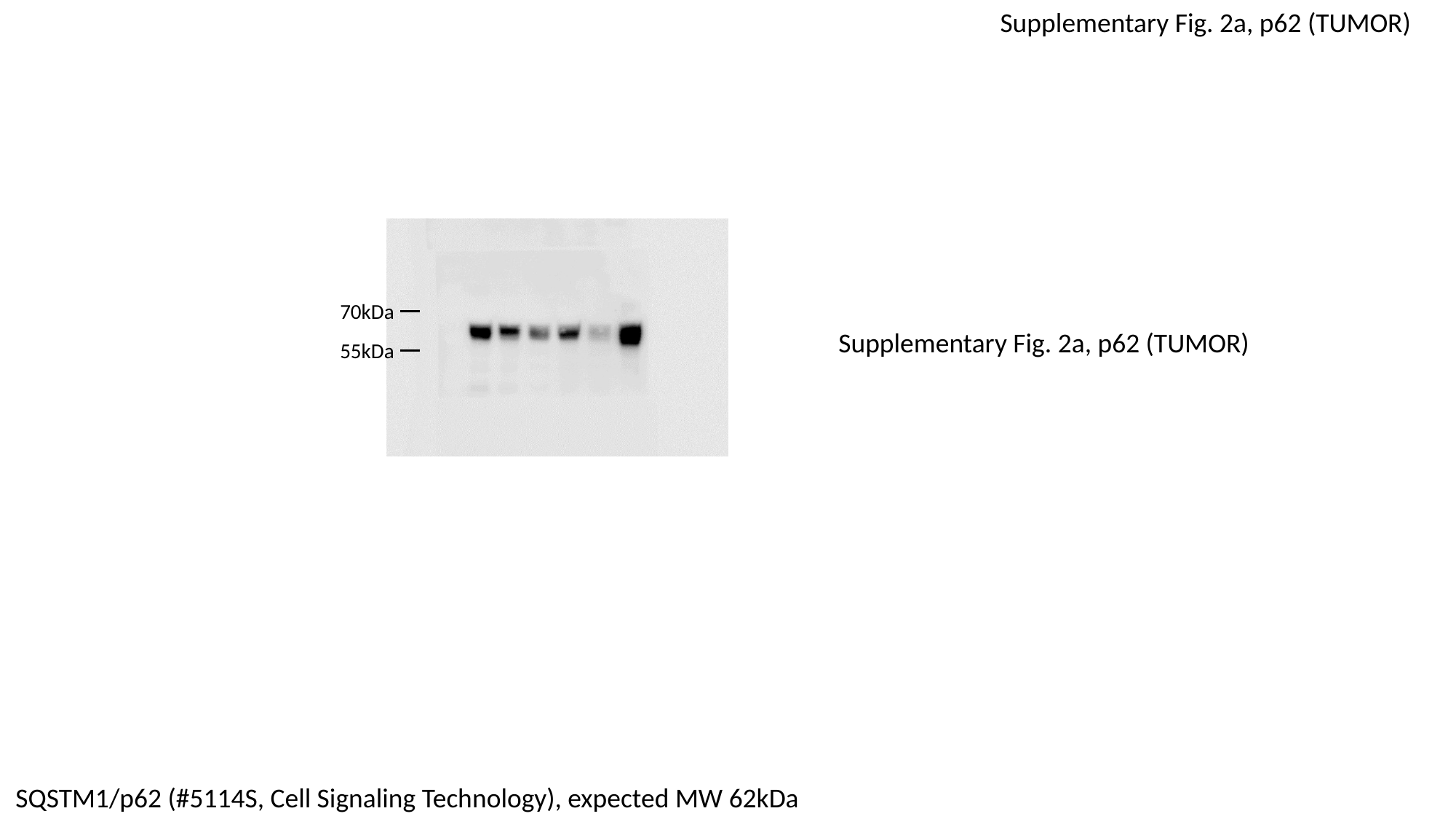

Supplementary Fig. 2a, p62 (TUMOR)
70kDa
Supplementary Fig. 2a, p62 (TUMOR)
55kDa
SQSTM1/p62 (#5114S, Cell Signaling Technology), expected MW 62kDa

## Slide 7
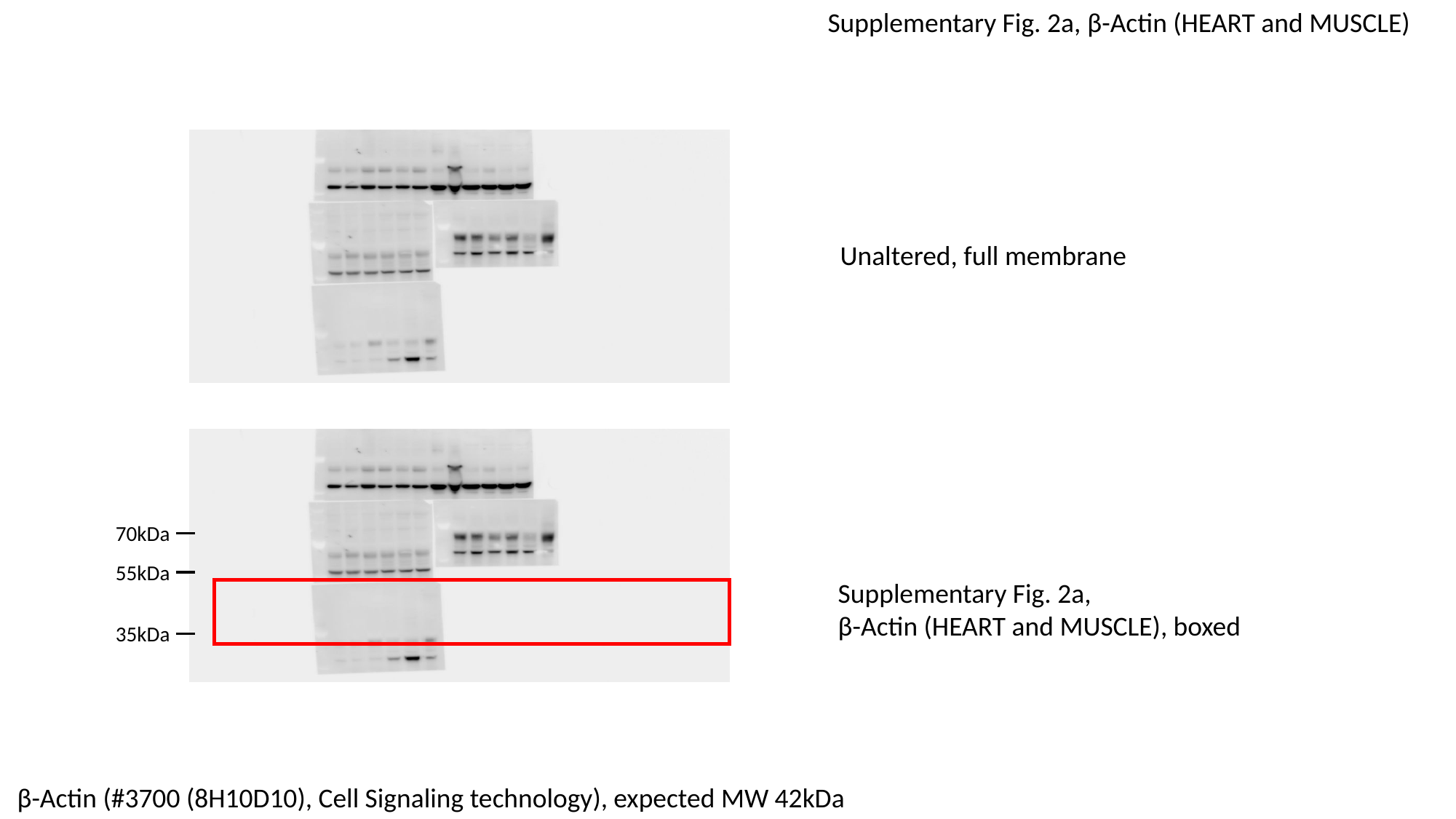

Supplementary Fig. 2a, β-Actin (HEART and MUSCLE)
Unaltered, full membrane
70kDa
55kDa
Supplementary Fig. 2a,
β-Actin (HEART and MUSCLE), boxed
35kDa
β-Actin (#3700 (8H10D10), Cell Signaling technology), expected MW 42kDa

## Slide 8
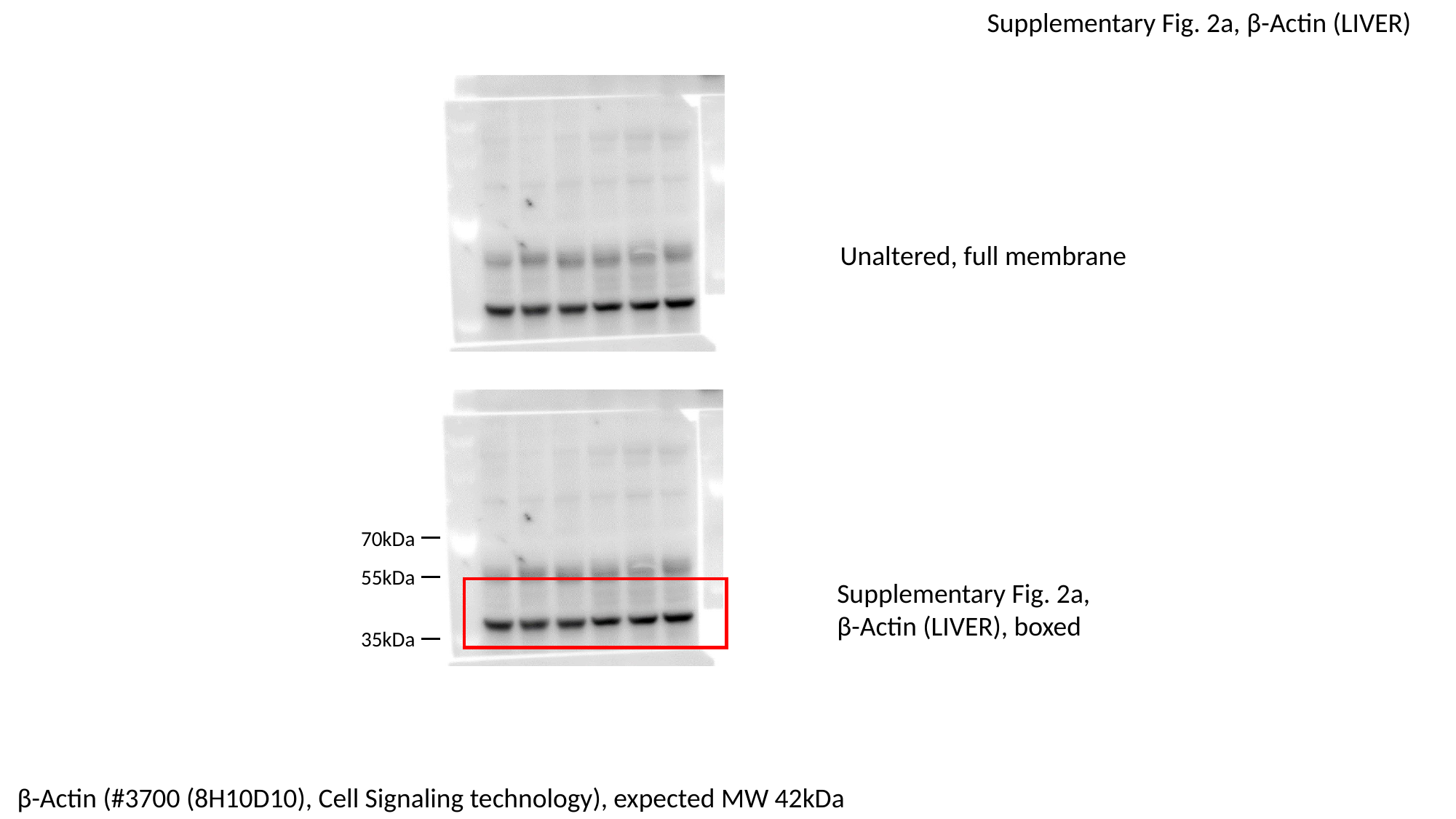

Supplementary Fig. 2a, β-Actin (LIVER)
Unaltered, full membrane
70kDa
55kDa
Supplementary Fig. 2a,
β-Actin (LIVER), boxed
35kDa
β-Actin (#3700 (8H10D10), Cell Signaling technology), expected MW 42kDa

## Slide 9
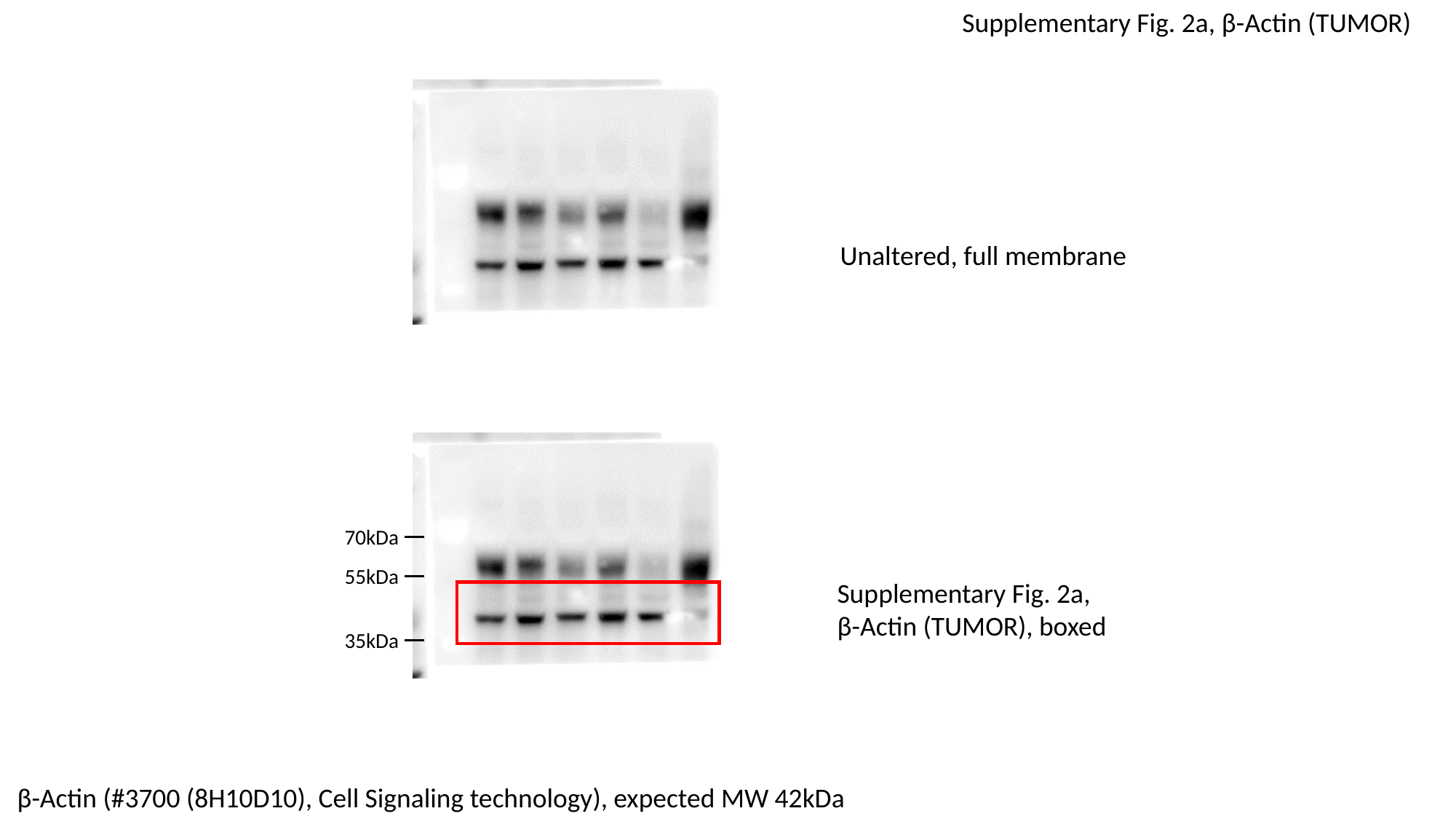

Supplementary Fig. 2a, β-Actin (TUMOR)
Unaltered, full membrane
70kDa
55kDa
Supplementary Fig. 2a,
β-Actin (TUMOR), boxed
35kDa
β-Actin (#3700 (8H10D10), Cell Signaling technology), expected MW 42kDa
