# Supplementary material for: Immunoprophylactic and immunotherapeutic control of hormone receptor-positive breast cancer
Source: Nat Commun. 2020 Jul 30;11:3819. doi: 10.1038/s41467-020-17644-0 (PMC7393498; doi:10.1038/s41467-020-17644-0)
Supplement: Supplementary file 6 — Source Data [file 41467_2020_17644_MOESM6_ESM.zip › Source Data/Suppl. Fig. 3 - Summary.pptx]

## Slide 1
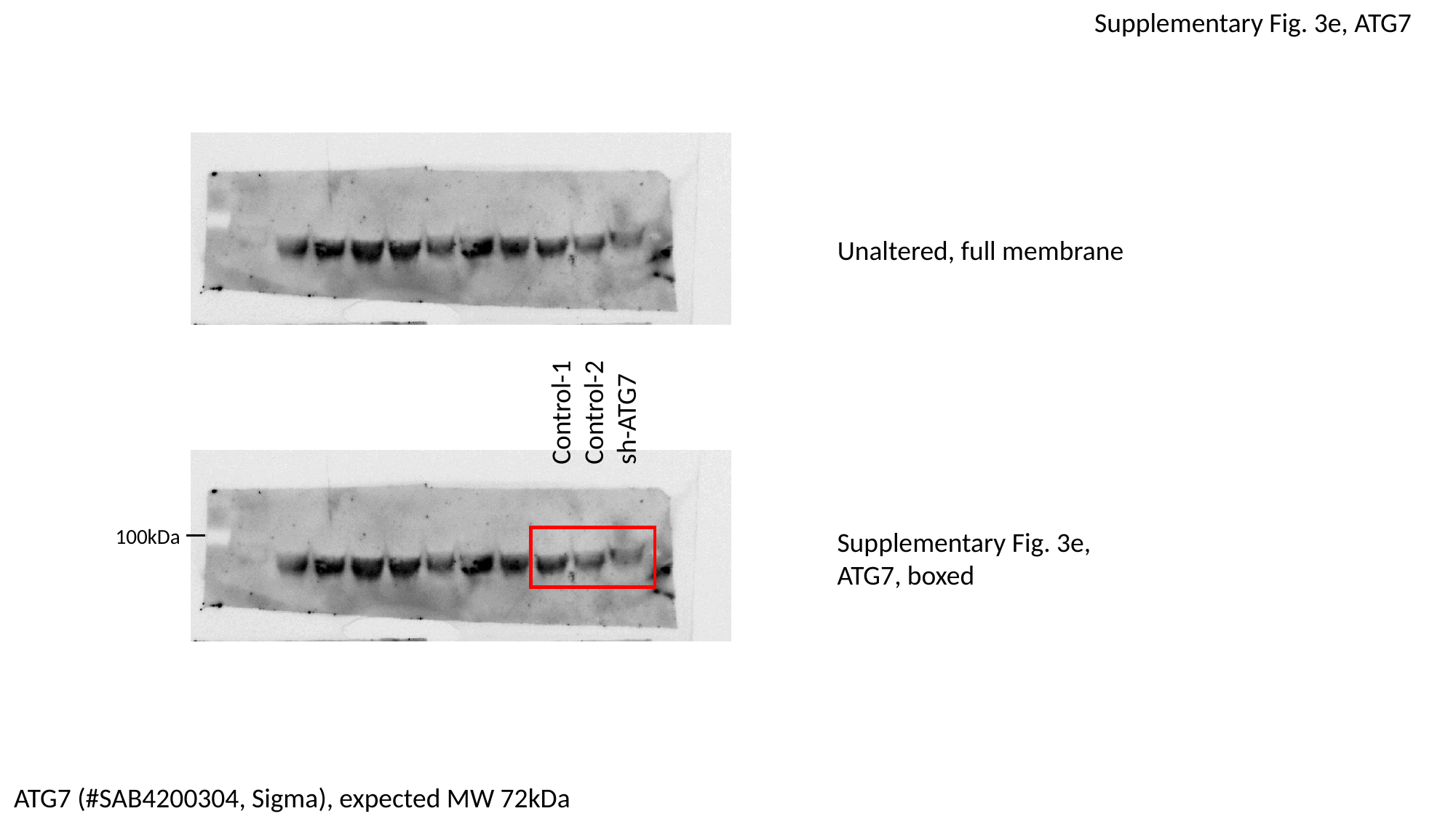

Supplementary Fig. 3e, ATG7
Unaltered, full membrane
Control-1
Control-2
sh-ATG7
100kDa
Supplementary Fig. 3e,
ATG7, boxed
ATG7 (#SAB4200304, Sigma), expected MW 72kDa

## Slide 2
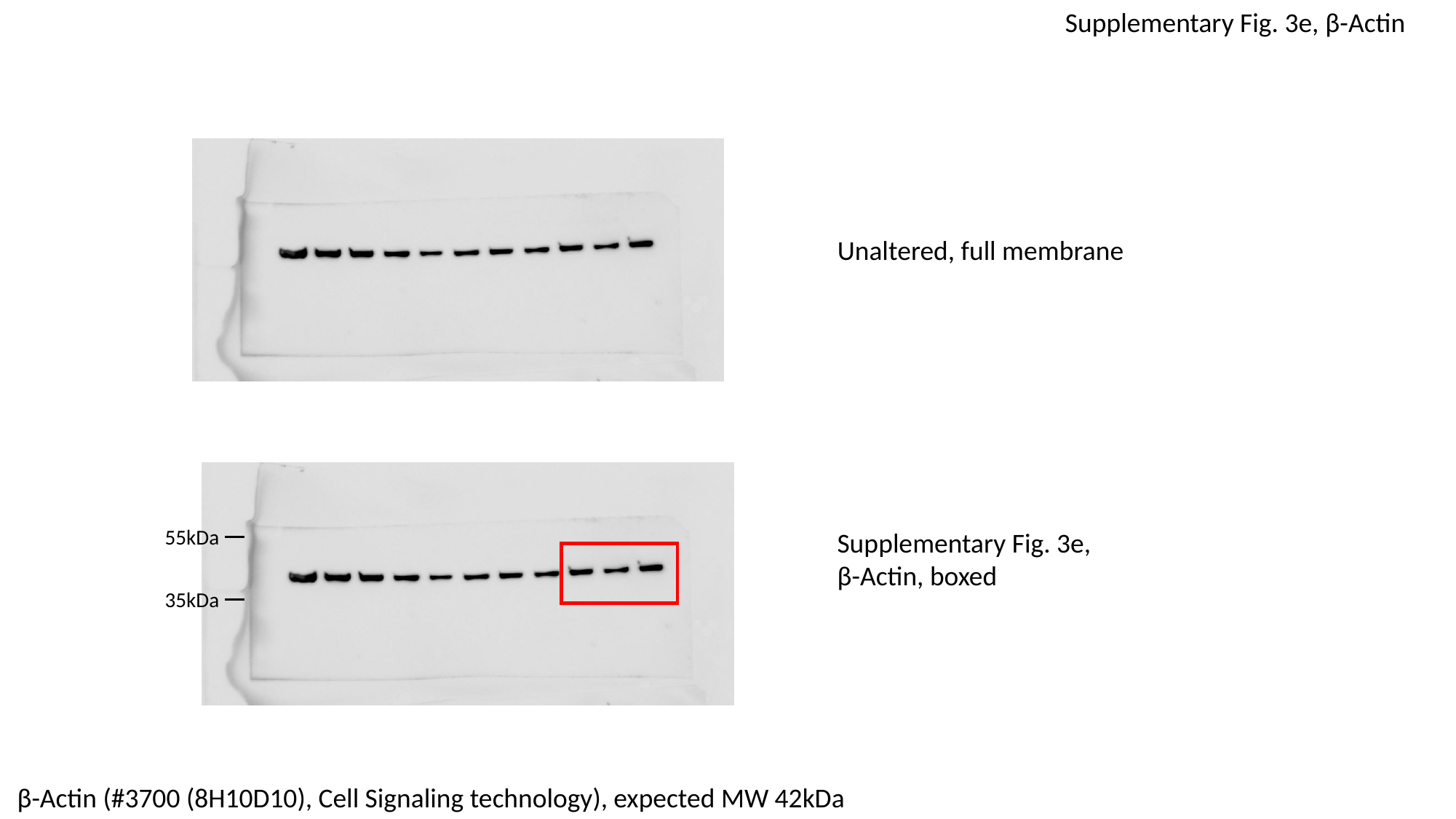

Supplementary Fig. 3e, β-Actin
Unaltered, full membrane
55kDa
Supplementary Fig. 3e,
β-Actin, boxed
35kDa
β-Actin (#3700 (8H10D10), Cell Signaling technology), expected MW 42kDa

## Slide 3
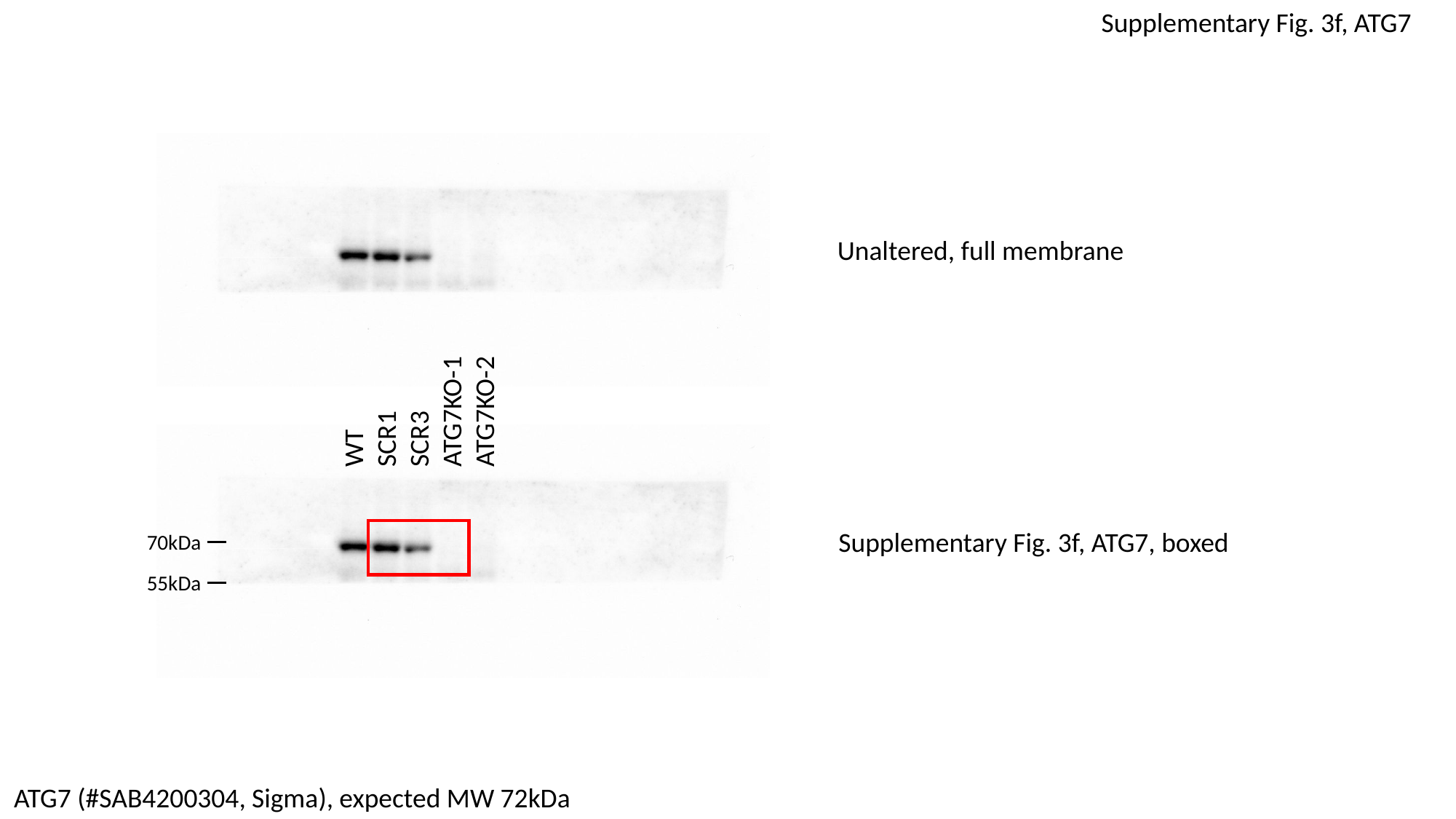

Supplementary Fig. 3f, ATG7
Unaltered, full membrane
WT
SCR1
SCR3
ATG7KO-1
ATG7KO-2
Supplementary Fig. 3f, ATG7, boxed
70kDa
55kDa
ATG7 (#SAB4200304, Sigma), expected MW 72kDa

## Slide 4
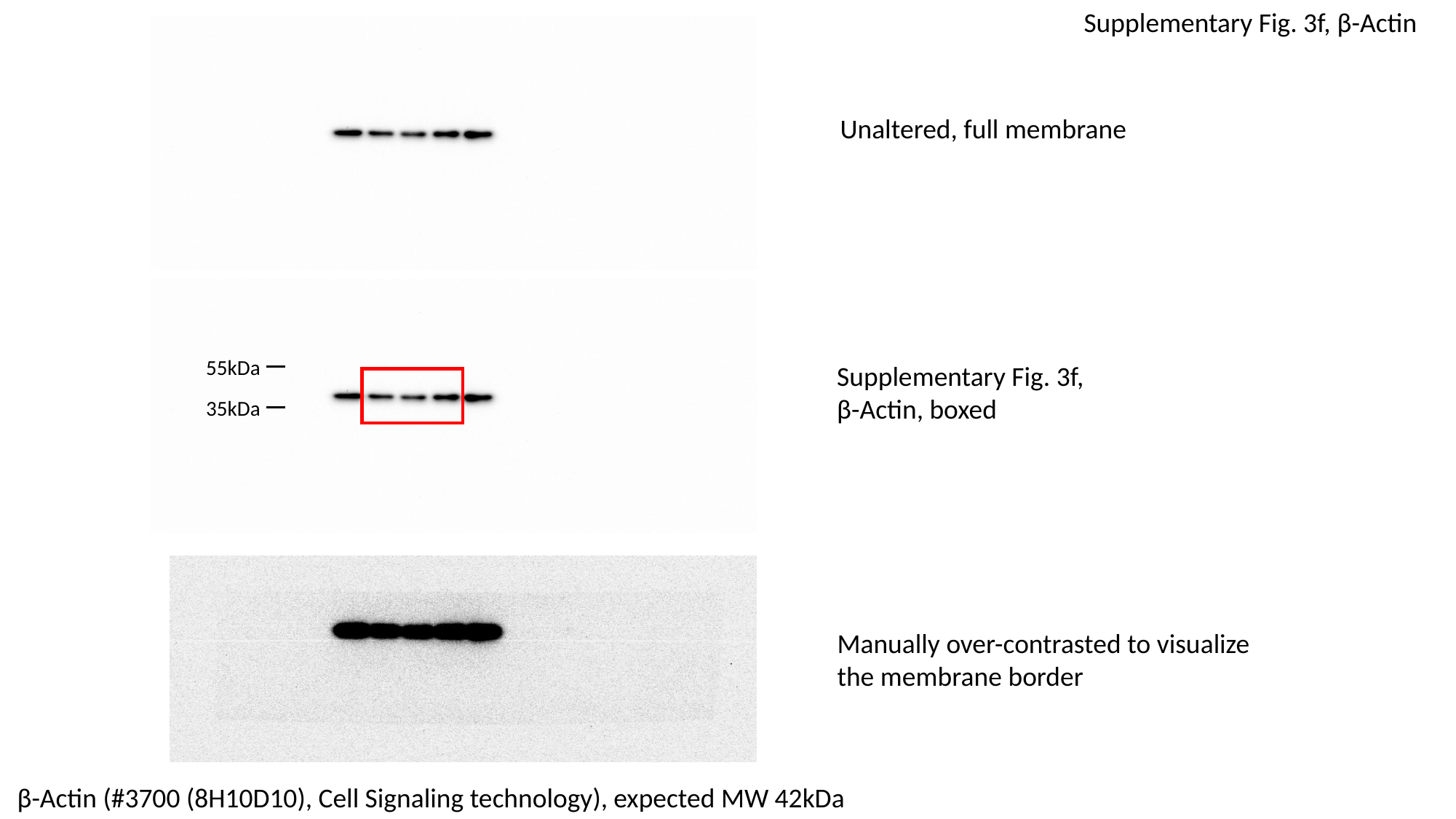

Supplementary Fig. 3f, β-Actin
Unaltered, full membrane
55kDa
Supplementary Fig. 3f,
β-Actin, boxed
35kDa
Manually over-contrasted to visualize
the membrane border
β-Actin (#3700 (8H10D10), Cell Signaling technology), expected MW 42kDa
