# Supplementary figures and images for: Immunoprophylactic and immunotherapeutic control of hormone receptor-positive breast cancer
Source: Nat Commun. 2020 Jul 30;11:3819. doi: 10.1038/s41467-020-17644-0 (PMC7393498; doi:10.1038/s41467-020-17644-0)

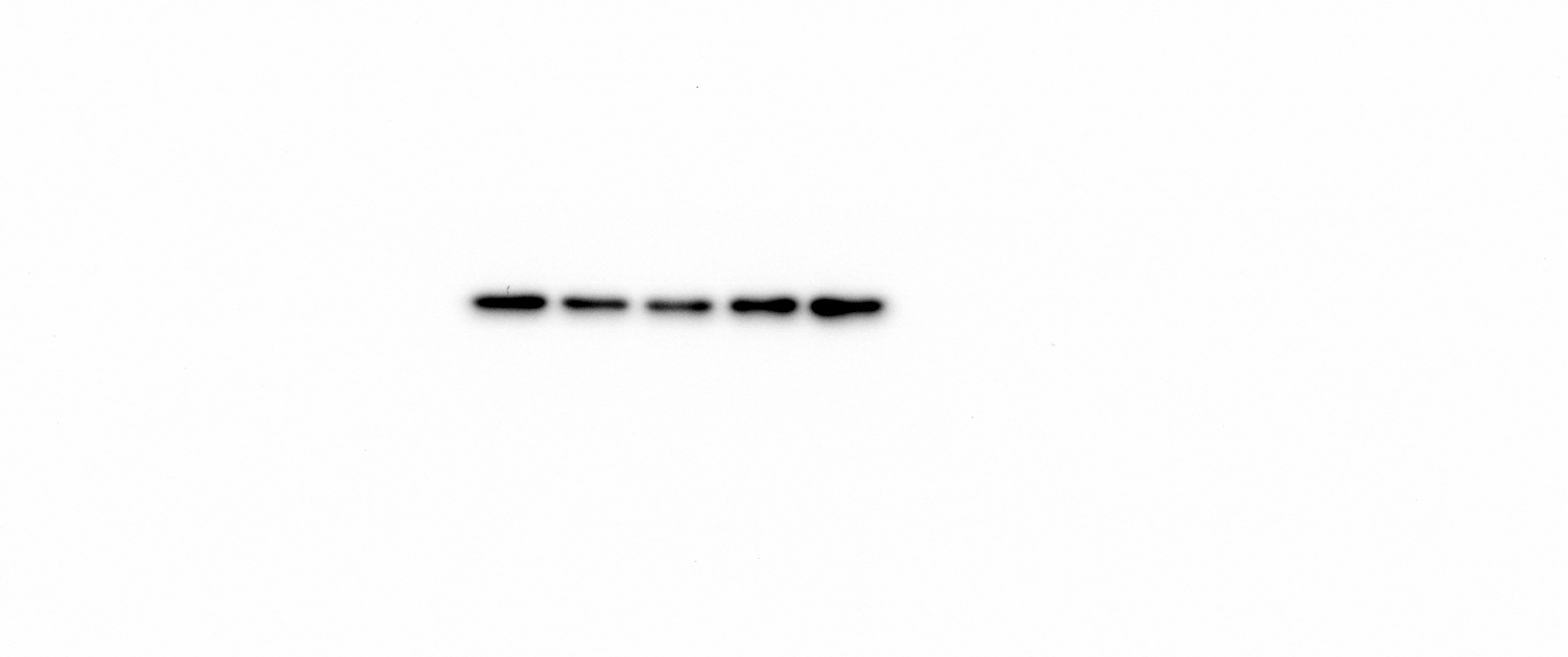

Supplement: Supplementary file 6 — Source Data [file 41467_2020_17644_MOESM6_ESM.zip › Source Data/Suppl. Fig. 3/f - ACTB.tif]

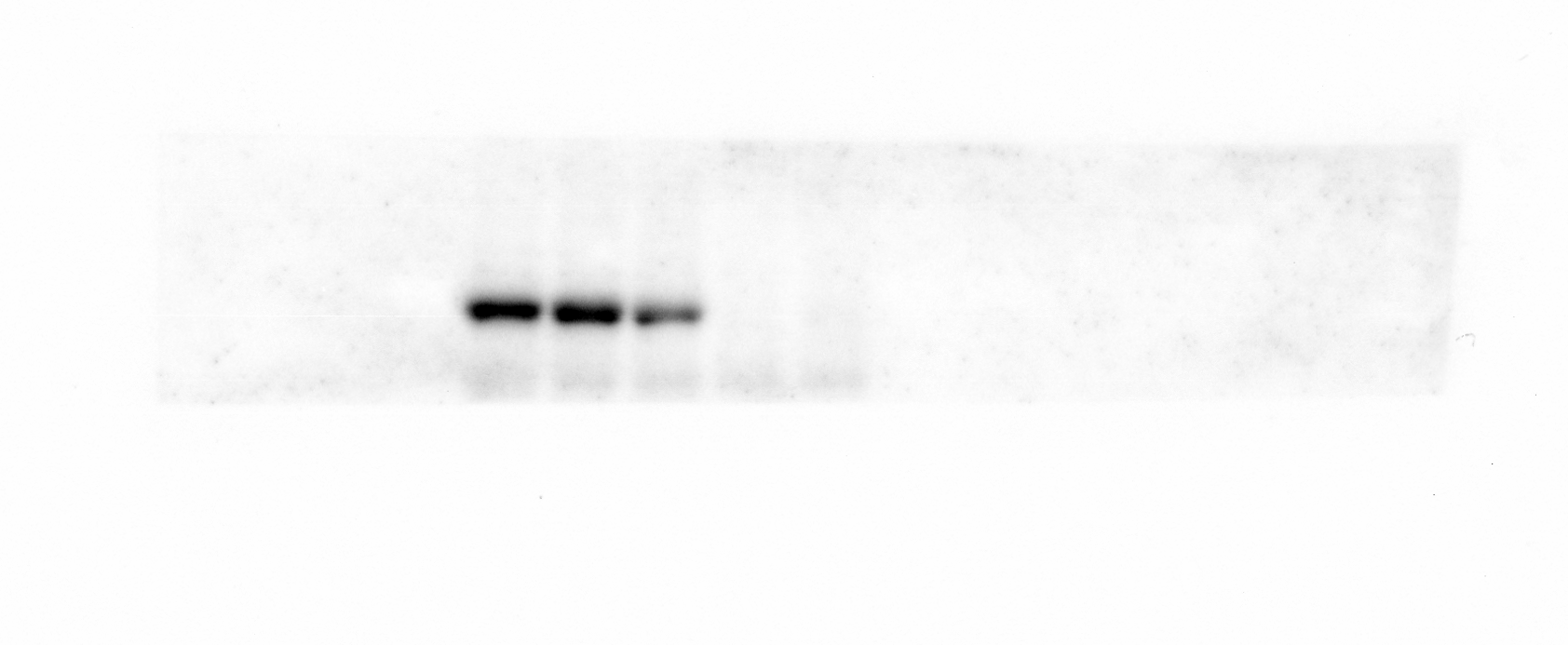

Supplement: Supplementary file 6 — Source Data [file 41467_2020_17644_MOESM6_ESM.zip › Source Data/Suppl. Fig. 3/f - ATG7.tif]
